# Prohibitin 2 is a key regulator of T cell proliferation, differentiation, and effector functions in vivo

**DOI:** 10.1038/s42003-026-10522-3

**Published:** 2026-07-20

**Authors:** Johannes F. Vogt, Carsten Merkwirth, Petra Adams-Quack, Elena Zurkowski, Emmanouill Stylianakis, David C. Uhlfelder, Thomas Michna, Assel Nurbekova, Ute Distler, Liliana Rojas-Charry, Hajime Yurugi, Sonja Reißig, F. Thomas Wunderlich, Krishnaraj Rajalingam, Stefan Tenzer, Axel Methner, Thomas Langer, Ari Waisman, Nadine Hövelmeyer

**Affiliations:** 1 Institute for Molecular Medicine Mainz, University Medical Center of the Johannes Gutenberg University Mainz, Mainz, Germany; 2 Institute for Genetics and Cologne Excellence Cluster on Cellular Stress Responses in Aging-Associated Diseases, University of Cologne, Cologne, Germany; 3 Research Center for Immunotherapy (FZI), University Medical Center of the Johannes Gutenberg University Mainz, Mainz, Germany; 4 Institute of Immunology, University Medical Center of the Johannes Gutenberg University Mainz, Mainz, Germany; 5 Cell Biology Unit, University Medical Center of the Johannes Gutenberg University Mainz, Mainz, Germany; 6 Max Planck Institute for Metabolism Research Cologne, Cologne, Germany; 7 Max Planck Institute for Biology of Ageing, Cologne, Germany

**Keywords:** T cells, Cell division

## Abstract

Prohibitin 2 (PHB2) is a highly conserved protein with essential roles in cell homeostasis and survival across different cell types. Previous studies have shown that the deletion of PHB2 results in an arrest in proliferation due to impaired mitochondrial function regulated by the dynamin-like GTPase OPA1. The function of PHB2 in immune cells remains unclear; however, some studies suggest that PHB2 plays a role in the cell membranes of B and T cells. In order to elucidate the role of PHB2 in immune cells, we generated PHB2-deficient T cells. Our findings reveal a pivotal role for PHB2 in the proliferation and differentiation of T cells. PHB2 deficiency inhibits T cell proliferation by inducing a cell cycle arrest at the G1 to S phase, thereby preventing the differentiation into effector T cells. Furthermore, in contrast to previous reports, T cells lacking PHB2 are more resistant to apoptosis. Metabolic analysis reveals that PHB2-deficient T cells fail to boost their energy production through glycolysis and oxidative phosphorylation upon activation, hindering their ability to sustain biosynthetic processes and to proliferate in response to activation.

## Abstract (web summary)

Mechanistic analysis reveals PHB2 as a key regulator of T cell function. Loss of PHB2 disrupts metabolic adaptation, impairs proliferation and differentiation, and alters survival pathways, highlighting its central role in T cell homeostasis.

## Introduction

Prohibitin 1 (PHB1) and prohibitin 2 (PHB2) are highly conserved proteins, ubiquitously expressed in essentially all cell types, ranging from murine embryonic fibroblasts, intestinal epithelial cells, β-cells to neurons^1–4^. PHB1 and PHB2 are interdependent on the protein level, and loss of one of them simultaneously leads to loss of the other^1^. Prohibitins exert and regulate a diverse range of cellular functions, including apoptosis, energy metabolism, proliferation, and senescence, based on their cellular location, either at the plasma membrane, mitochondria, or the nucleus^5,6^. In mitochondria, both proteins assemble at the inner mitochondrial membrane and form a supra-macromolecular structure that regulates the mitochondrial genome, modulates mitochondrial dynamics, morphology, biogenesis, and the mitochondrial intrinsic apoptotic pathway^7,8^.

While mitochondrial Phbs are always present in all cell types studied so far, a considerable proportion of PHB1 and PHB2 were shown to be expressed on the surface of T cell receptor (TCR) activated T cells in mice and humans^6,9^. Specifically, PHB1 and PHB2 are highly expressed on the surface of Th17 cells, contributing to the differentiation into these effector cells^10^. Engagement of surface-expressed PHB1/2 with Vi polysaccharide leads to the inhibition of the CRAF-MAPK signaling cascade that controls the plasticity of Th17 cells, although this interaction does not affect the proliferative capacity of T cells^10^. The knockdown of prohibitins in Kit255 cells, a T cell leukemia cell line, led to a reduced mitochondrial membrane potential^9^. This suggests that prohibitins on the cell surface of activated T cells are involved in TCR-mediated signaling^6^. Although these in vitro studies strongly indicate a role of prohibitins in T cell function, in vivo evidence is still lacking.

In mice, it was shown that loss of the PHB complex during development and mice with a brain-specific deletion of *Phb2*, using Nestin-Cre, results in embryonic lethality^11^. Postnatal knockout of PHB2 in the forebrain using *CaMKIIα*-driven Cre expression leads to neuronal loss and death at around 17 weeks of age^4^. To determine the function of PHB2 in T cells, we examined mice with a T cell-specific PHB2-deficiency and found that PHB2 is essential for T cell homeostasis, proliferation, function, and T cell differentiation. At steady state, the T cell numbers of mice lacking PHB2 were significantly reduced in all secondary lymphoid organs tested. Even though PHB2-deficient T cells still show typical early TCR activation signs, such as increased expression of CD25 and CD69, these naive T cells are unable to differentiate into effector T cells and to secrete effector cytokines upon activation. Furthermore, T cells lacking PHB2 exhibit a significant proliferative defect resulting from cell cycle arrest at G1 to S-phase. This is due to a defect in initiating DNA replication following TCR stimulation, which is accompanied by a significantly reduced expression of numerous proteins crucial for T cell proliferation. Thus, our study highlights the significance of PHB2 for T cell functions in vivo.

## Results

### PHB2 is essential for T cell homeostasis in vivo

To study the role of PHB2 in T cells in vivo, we crossed conditional *Phb2*^*F/F* 1^ mice with CD4-Cre^12^ mice, resulting in the deletion of PHB2 in all T cells from the CD4^+^CD8^+^ double-positive progenitor state during thymic development. For brevity, these mice are referred to as *Phb2*^*TKO*^.

Even though CD4-Cre expression starts in the double-positive state of T cell development in the thymus, flow cytometric analysis revealed no changes in thymocyte cell numbers or developmental stages, including CD4^-^CD8^-^ DN, CD4^+^ SP, CD8^+^ SP, or CD4^+^CD8^+^ DP thymocytes, in *Phb2*^*TKO*^ mice compared to littermate controls (Supplementary Fig. 1A, B). Furthermore, earlier developmental stages (DN1-DN4) were also unaffected in *Phb2*^*TKO*^ mice compared to littermate controls (Supplementary Fig. 1C). To understand the reason why thymic T cells were unaffected by the loss of PHB2, we examined CD4-Cre-mediated deletion of PHB2 protein by Western blot analysis of MACS-purified CD4^+^ thymic T cells. Our results showed that PHB2 protein was still present at equal amounts in *Phb2*^*TKO*^ thymocytes compared to control cells, suggesting a prolonged half-life of PHB2 protein (Supplementary Fig. 1E).

**Fig. 1:**
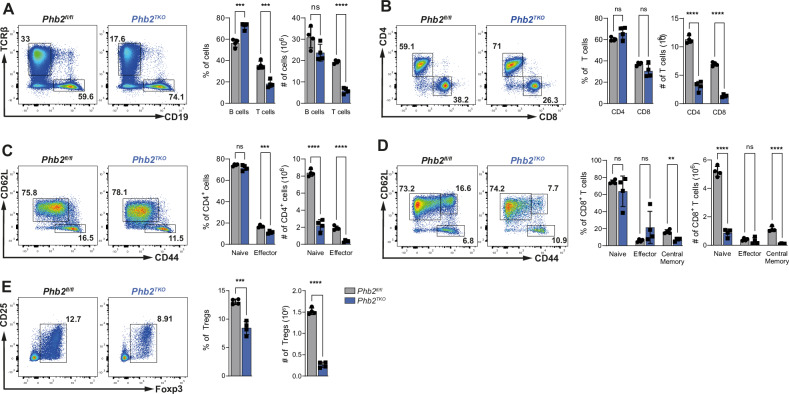
Prohibitin 2 is essential for T cell homeostasis in vivo. **A** Flow cytometric (left) and statistical analysis (right) of live B cells (CD19^+^) and T cells (TCRβ^+^) **B** Flow cytometric (left) and statistical analysis (right) of live CD4^+^ and CD8^+^ T cells **C** Flow cytometric (left) and statistical analysis (right) of live CD4^+^ naïve (CD62L^+^ CD44^low^) and CD4 effector (CD62L^-^ CD44^high^) T cells **D** Flow cytometric (left) and statistical analysis (right) of live CD8^+^ naïve (CD62L^+^ CD44^low^), effector CD8^+^ (CD62L^-^ CD44^high^), and central memory CD8+ (CD62L^+^ CD44^high^) T cells **E** Flow cytometric (left) and statistical analysis (right) of live Treg cells (Foxp3^+^) **A**-**E**: Data is representative of at least three independent experiments with *n* = 4. Bar graphs show means +/- SDs and single values. Statistical significance was calculated using an unpaired two-tailed *t*-test with Holm-Šidák correction for multiple comparisons. **p ≤ 0.01 ***p ≤ 0.001, ****p ≤ 0.0001.

The total cell counts of the thymus, spleen, lymph node (LN), and mesenteric lymph nodes (mLN) were comparable between *Phb2*^*TKO*^ mice and littermate control mice, with no significant differences observed (Supplementary Fig. 1A). Notably, the percentage and total cell count of TCRβ^+^ T cells in the spleen were significantly reduced by approximately 50% in *Phb2*^*TKO*^ mice compared to littermate controls (Fig. 1A). Conversely, the percentage of CD19^+^ B cells in the spleen was significantly increased, although the total cell count remained unchanged (Fig. 1A). Furthermore, the ratio of CD4^+^ and CD8^+^ T cells in the spleen was unchanged, but the total cell count of CD4^+^ as well as CD8^+^ T cells was significantly reduced in *Phb2*^*TKO*^ mice compared to littermate controls (Fig. 1B). Comparable effects were observed in both LN and mLN (Supplementary Fig. 1C). Furthermore, when CD4^+^ T cells were analyzed for the expression of activation markers, we again observed a significant reduction when subdividing the CD4^+^ T cells into naïve (CD62L^high^CD44^low^) and memory/effector T cells (CD62L^low^CD44^high^) (Fig. 1C). The total cell counts of naïve and central memory CD8^+^ T cells were also significantly reduced, whereas the number of effector CD8^+^ T cells remained unchanged (Fig. 1D). To test the deletion efficiency in these populations, we performed Western blot analysis of FACS sorted naïve CD4^+^ and CD8^+^ T cells. This analysis revealed a complete deletion of PHB2 protein (Supplementary Fig. 1E). In contrast, the CD4^+^ and CD8^+^ effector T cells of *Phb2*^*TKO*^ mice contained some residual PHB2 protein (Supplementary Fig. 1E).

Another T cell subset that is affected by CD4-Cre-mediated deletion is Foxp3^+^ regulatory T (Treg) cells. Flow cytometric analysis of splenic Treg cells also showed a significant reduction in this cell population in percentage as well as total cell count compared to controls (Fig. 1E). To assess the deletion efficiency of PHB2 in CD4^+^CD25^+^ T cells, we performed Western blot on sorted cells. As expected, this analysis revealed an almost complete loss of PHB2 protein in Treg cells (Supplementary Fig. 1F). Furthermore, consistent with previous findings^1^, the deletion of *PHB2* also led to the loss of PHB1 protein (Supplementary Fig. 1G).

In summary, the deletion of PHB2 in T cells results in a dramatic decrease of all T cell subsets at steady state in peripheral lymphoid organs. These results show that the loss of PHB2 in T cells prevents appropriate T cell expansion in the periphery, while T cell development in the thymus appears to be normal.

### Prohibitin 2 is essential for in vivo effector T cell function

To assess the function of PHB2-deficient T cells in vivo, we induced experimental autoimmune encephalomyelitis (EAE). EAE is a mouse model of multiple sclerosis and is highly dependent on T cell effector functions. This autoimmune reaction is activated by injecting the MOG peptide p35-55 emulsified in CFA, which activates autoimmune T cells and promotes their migration into the central nervous system^13^. This process leads to CD4^+^ T cell-mediated destruction of neuronal myelin sheaths in the central nervous system.

**Fig. 2:**
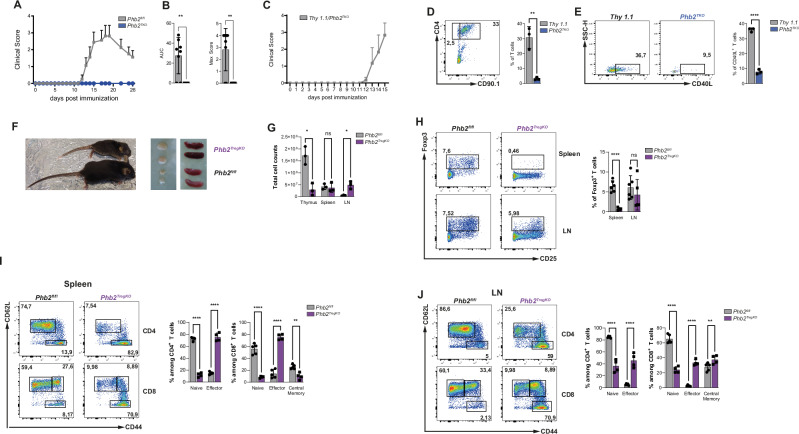
PHB2 is essential for T cell effector function. **A** Clinical signs of EAE are shown as mean clinical disease scores ± SD. (*n* = 5-7/genotype). **B** Area Under the Curve (AUC) and maximum clinical scores of (**A**). *n *= 7-8, Mann-Whitney test ± SD (**C**) Clinical signs of EAE of bone marrow chimera of *Thy1.1*^*+*^ and *Phb2*^*TKO*^ mice. *n* = 3/genotype (**D**) Flow Cytometric (left) and statistical analysis (right) of CD4^+^ CD90^+^ T cells from the CNS of mice from C (**E**) Flow Cytometric (left) and statistical analysis (right) of CD40L^+^ T cells from the CNS of mice from C (**F**) Representative picture of 29-day-old control and *Phb2*^*TregKO*^ mice and their spleen and inguinal lymph nodes (scale bars = 5 mm) **G** Total cell count of thymus, spleen, and LN of 29-day-old control and *Phb2*^*TregKO*^ mice. *n* = 3 **H** Flow Cytometric (left) and statistical analysis (right) of Treg cells (Foxp3^+^). *n* = 4–6 mice/genotype. **I**, **J** Flow Cytometric (left) and statistical analysis (right) CD4^+^ naïve (CD62L^+^ CD44^low^) and CD4^+^ effector (CD62L^-^ CD44^high^) T cells and naïve CD8^+^ (CD62L+ CD44^low^), CD8^+^ effector (CD62L^-^ CD44^high^), and central memory CD8^+^ (CD62L^+^ CD44^high^) T cells in spleen (**I**) and LN (**J**) Data is representative of three (**A**) one (**C**–**E**), or pooled from two (**G**–**J**) independent experiments. Bar graphs show means +/- SDs and single values (**D**, **E**, **G**–**J**). Statistical significance was calculated using an unpaired two-tailed t-test with Holm-Šidák correction for multiple comparisons. *p < 0.05, **p ≤ 0.01, ***p ≤ 0.001, ****p ≤ 0.0001.

We found that mice lacking PHB2, specifically in T cells, were completely resistant to EAE compared to control animals (Fig. 2A, B). In order to rule out a paracrine effect, or effects observed due to significantly decreased T cell numbers in PHB2^TKO^ mice, we generated bone marrow (BM) chimeras. We isolated BM cells from Thy1.1 control and Thy1.2 *Phb2*^*TKO*^ mice, and these cells were injected (in a ratio of 1:1) into Ly5.1 mice that were previously mildly irradiated. Ten weeks later, EAE was induced in these animals. The disease progressed was measured, and the mice were analyzed at the peak of the disease on day 15 by flow cytometry (Fig. 2C). This analysis revealed that PHB2-deficient CD4^+^ T cells almost were absent in the central nervous system (CNS) of sick mice, which only contained Thy1.1 WT T cells (Fig. 2D). Furthermore, the MOG recall-assay, an assay for MOG specific auto-immune T cells, showed a significant reduction in CNS infiltration by MOG-specific PHB2-deficient CD4^+^ T cells (Fig. 2E).

These results show that T cells lacking PHB2 are incapable of inducing disease and of responding to the MOG peptide used for the immunization. Given that *Phb2*^*TKO*^ mice are healthy until the age of analysis (8-12 weeks of age and under SPF conditions), yet their T cells are incapable of mounting an immune response, we decided to cross *PHB2*^*fl/fl*^ mice with Foxp3-Cre mice to investigate potential defects in PHB2-deficient regulatory T cells.

*Phb2*^*TregKO*^ mice exhibited clear signs of autoinflammation and had to be sacrificed between 22–32 days after birth due to severe signs of inflammation. Macroscopically, *Phb2*^*TregKO*^ mice were smaller than control mice and displayed scaly skin, crusting of the eyelids, ears and tail, signs of a scurfy phenotype (Fig. 2F)^14^. These mice showed enlarged lymph nodes (LN) while the spleens were similar in size compared to those of control mice (Fig. 2F). Consistent with its size, the total cell counts of the spleen remained unchanged when comparing *Phb2*^*TregKO*^ with control mice (Fig. 2G). However, the peripheral LN of *Phb2*^*TregKO*^ mice displayed a marked increase in total cell numbers (Fig. 2G). A detailed analysis of the thymus revealed a dramatic reduction in the total cell count (Fig. 2G).

Flow cytometric analysis revealed a significant reduction in the percentage of Treg cells in the spleen, while the % in the LN remained unchanged (Fig. 2H). Additionally, analysis of effector T cells showed a dramatic increase in CD4^+^ and CD8^+^ effector T cells in both the spleen and LN of *Phb2*^*TregKO*^ mice compared to control mice (Fig. 2I, J). Together, these results resemble the scurfy phenotype, an X-linked recessive mutation that leads to a loss of Treg function and subsequent fatal immune dysfunction^14^. These findings collectively underscore the critical importance of PHB2 for the function of T reg cells.

### PHB2 regulates proteins that are crucial for T cell proliferation

**Fig. 3:**
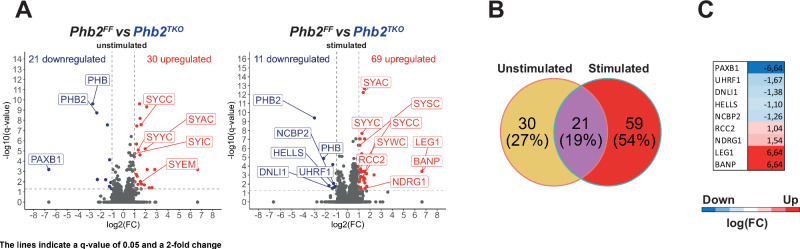
PHB2 regulates proteins that are essential for proliferation. **A** Volcano plots of mass spectrometric analysis of unstimulated (left) and α-CD3/α-CD28 stimulated (right) naïve CD4^+^ T cells from control and *Phb2*^*TKO*^ mice. **B** Venn diagram of significantly regulated peptides in unstimulated and stimulated naïve CD4^+^ T cells from control and *Phb2*^*TKO*^ mice. **C** Manually annotated list of significantly regulated peptides with a negative effect on proliferation.

To obtain an unbiased view of the effect of PHB2 deficiency on T cells, we performed a comparative proteomic analysis of MACS-purified splenic naïve CD4^+^ T cells from *Phb2*^*TKO*^ and control mice at steady state and after α-CD3/α-CD28 stimulation for 24 h (Fig. 3).

At steady state, T cells from *Phb2*^*TKO*^ mice were significantly different from T cells from control mice. In unstimulated PHB2-deficient naïve CD4^+^ T cells, 21 peptides were significantly downregulated and 30 peptides upregulated compared to naïve control T cells (Fig. 3A, left).

After 24 h of α-CD3/α-CD28 TCR stimulation, eleven peptides were significantly downregulated, and 69 peptides were upregulated in PHB2-deficient T cells compared to control T cells (Fig. 3A, right). Of these, 21 peptides were significantly altered under both unstimulated and stimulated conditions (Fig. 3B, Supplementary Fig. 2A). Surprisingly, the changes in expression levels of these 21 peptides were identical under both conditions, suggesting that the stimulation itself did not affect the expression of these peptides (Supplementary Fig. 2A). This analysis also revealed the loss of both PHB2 and PHB1 in the transgenic T cells compared to control mice (Supplementary Fig. 2A). At steady state and after stimulation, annexin A2 (Anxa2), a protein that has already been reported to be directly associated with PHB1 and PHB2^15^, was upregulated in PHB2-deficient T cells compared to control cells (Supplementary Fig. 2A). After stimulation with α-CD3/α-CD28, the ATP-dependent Zinc metalloprotease YME1L1 was downregulated in PHB2-deficient T cells. YME1L1, in conjunction with OMA1, cleaves OPA-1, resulting in a disbalance in the S-OPA1/L-OPA1 ratio. This imbalance is responsible for the fragmented mitochondrial phenotype observed under PHB2 deficiency^1^^16^. YME1L1 downregulation in PHB2-deficient T cells in comparison to control T cells suggests a potential compensatory mechanism (Supplementary Fig. 2A).

This data set was further analyzed using the Ingenuity Pathway Analysis (IPA). This pathway analysis revealed no significant regulated pathways related to proliferation, metabolism, or T cells in general. However, tRNA charging was significantly upregulated in PHB2-deficient T cells under steady state and after stimulation in comparison to control T cells (Supplementary Fig. 2B). This pathway consists of tRNA synthetases, ligases that mediate the loading of tRNA with amino acids^17^. Furthermore, IPA’s functional analysis revealed that PHB2-deficient T cells exhibit an increase in the expression of peptides associated with T cell activation and T cell development when compared to control cells (Supplementary Fig. 2C).

Next, we manually annotated our data set and found a set of peptides associated with cell proliferation. We found nine differentially expressed peptides that are associated with proliferation and cell cycle progression (Fig. 3C), five of those with indication for a direct role in T cell proliferation (Supplementary Table 1). All these peptides were regulated in a way that they would negatively affect the proliferation of T cells. Out of these proteins, HELLS, a helicase that establishes DNA methylation patterns, has already been reported to affect peripheral lymphocyte homeostasis and T cell proliferation^18^, but is not associated with prohibitins.

Collectively, these data suggest a pro-proliferative role of PHB2 in T cells, as indicated by the downregulation of proteins essential for proliferation.

### PHB2 is essential for proper T cell proliferation

**Fig. 4:**
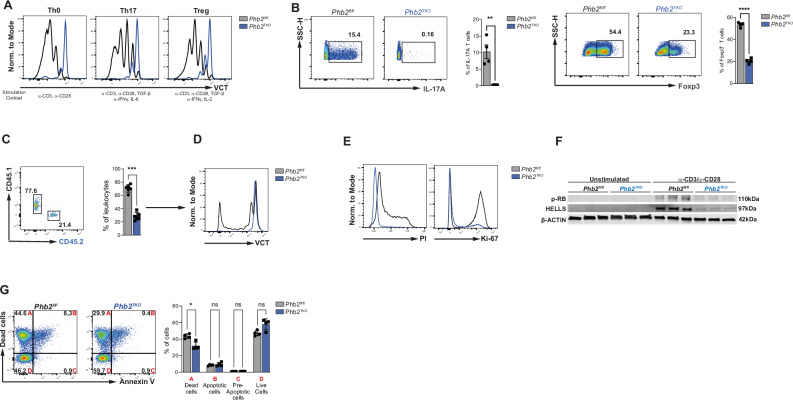
PHB2 deletion results in defects in T cell proliferation and differentiation. **A** Representative histogram illustrating CellTrace Violet (VCT) dilution as a measure of T cell proliferation of live CD4^+^ naïve T cells cultured with Th0 (α-CD3/α-CD28), Th17 (α-CD3/α-CD28, TGF-β, α-IFNg, IL-6), and Treg cells (αCD3-αCD28, TGF-β, α-IFNg, IL-2), under inducing conditions for 96 h (*n* = 4/genotype) (**B**) Flow cytometric (left) and statistical analysis (right) of IL-17A and Foxp3 expression of cells from A (*n *= 4/genotype) (**C**) Flow cytometric (left) and statistical (right) analysis of the co-transfer of CD45.1^+^ control and CD45.2^+^ PHB2-deficient naïve CD4^+^ T cells into RAG2^-/-^ mice (*n *= 5/genotype) (**D**) Representative histogram of CellTrace Violet (VCT) dilution on CD4^+^ T cells from B (*n* = 5/genotype) (**E**) Representative histogram of propidium iodide (PI) expression (left panel) as measured by flow cytometry of CD4^+^ T cells expression after 24 h α-CD3/α-CD28 stimulation (*n* = 3/genotype). Right panel: Representative histogram of Ki-67 expression after 24 h α-CD3/α-CD28 stimulation (*n* = 3/genotype) **F** Western blot analysis of p-Rb and HELLS expression of unstimulated and 24 h α-CD28/α-CD28 stimulated naïve CD4^+^ T cells (*n* = 4/genotype) (**G**) Flow cytometric (left) and statistical (right) analysis of apoptotic CD4^+^ T cells after 24 h of in vitro stimulation with α-CD3/α-CD28 (*n* = 4). (Annexin V-/PI -: viable, non-apoptotic CD4 + T cells, Annexin V + /PI-: early apoptotic CD4 + T cells; Annexin V + /PI + : late apoptotic/necrotic cells; Annexin V-/PI + : Dead cells). Data are representative of at least three independent experiments (**A**, **B**, **E**, **F**) and one (**C**, **D**) experiment. Analyses were performed on 8-12-week-old mice. Bar graphs show means +/- SDs and single values (**B**, **C**, **F**). Statistical significance was calculated using an unpaired two-tailed t-test with Holm-Šidák correction for multiple comparisons. *p < 0.05, **p ≤ 0.01, ***p ≤ 0.001, ***p ≤ 0.0001.

To determine if the downregulation of proliferation-associated proteins observed in the proteomics data set from PHB2-deficient T cells leads to a proliferative defect and a subsequent reduction in all peripheral T cell subsets, we labeled naïve CD4^+^ T cells with a violet cell tracer (VCT) and activated the cells via the TCR. In addition, we added cytokine cocktails to induce their differentiation to Th1, Th17, or Treg cells. We detected a profound proliferative defect of PHB2-deficient T cells under Th0 conditions (α-CD3/α-CD28 stimulation), Th17, and Treg cells conditions (Fig. 4A). To better characterize Th cell effector function, we investigated cytokine secretion by CD4^+^ T cells from *Phb2*^*TKO*^ and control mice. Differentiation of naïve CD4^+^ T cells into Th17 and iTregs for four days revealed a significant reduction of IL-17A^+^ and Foxp3^+^ cells in PHB2-deficient T cells compared to control cells (Fig. 4B).

Since there was still a small proportion of T cells isolated from PHB2-deficient mice that were able to proliferate, as seen in Fig. 4A and Supplementary Fig. 3A left, we analyzed whether these cells possibly escaped CD4-Cre-mediated deletion of PHB2. Therefore, CFSE^low^ T cells (that strongly proliferated) and CFSE^high^ T cells (that did not proliferate) were FACS sorted for Western blot analysis of PHB2 expression. This analysis revealed that indeed, the few T cells that were able to proliferate still expressed PHB2 and escaped Cre-mediated recombination (Supplementary Fig. 3A right). Thus, PHB2 deficiency strongly suppresses T cell proliferation in vitro. To investigate whether PHB is upregulated during T cell activation and proliferation, we isolated wild-type T cells and left them either unstimulated or treated them with different T cell activating stimuli. As shown in Supplementary Fig. 3B, wild-type T cells upregulated PHB2 expression in response to activation, indicating that PHB2 plays a role in T cell activation and proliferation. These data are in line with previous reports, where PHB2 was proven to be essential for proliferation in other cells^1,19^.

To evaluate whether the proliferative defect observed in vitro can also be translated to in vivo conditions, we co-transferred MACS-sorted VCT-labeled naïve CD4^+^ T cells with different congenic markers (control T cells CD45.1^+^) and *Phb2*^*TKO*^ T cells CD45.2^+^ in a ratio of 1:1 into *RAG2*^*-/-*^ mice. After six days, T cells in the spleens of *RAG2*^*-/-*^ mice were analyzed by flow cytometry. Control CD45.1^+^ T cells made up 77,6% of all CD90.2^+^ T cells found in the spleens of *RAG2*^*-/-*^ mice, while PHB2-deficient CD45.2^+^ T cells made up 21,4% (Fig. 4C). VCT staining showed that control CD45.1^+^ T cells successfully proliferated in *RAG2*^*-/-*^ mice (Fig. 4D), while CD45.2^+^ PHB2-deficient T cells did not proliferate (Fig. 4D).

In order to determine whether the significant decrease in peripheral T cells in PHB2-deficient mice is attributed to a block at a specific stage of the cell cycle, we performed propidium iodide staining of naïve CD4^+^ T cells that were stimulated for three days with α-CD3/α-CD28. This analysis revealed a complete block of PHB2-deficient T cells to initiate DNA replication (Fig. 4E, left). Furthermore, the absence of Ki-67 staining in PHB2-deficient T cells after three days of stimulation suggests a block in cell cycle progression at the G1 to S-phase transition, since Ki-67 is upregulated upon entry into the S-phase of the cell cycle^20^ (Fig. 4E, right).

Our proteomics data (Fig. 3) revealed that proliferation-associated proteins are downregulated in stimulated PHB2-deficient T cells (Fig. 4C). Interestingly, one of the identified proteins, HELLS, together with UHRF1, was reported to be regulated by the Rb/E2f family, contributing directly to retinoblastoma tumorigenesis^21^. Concomitantly, UHRF, which also establishes DNA methylation patterns, was also significantly downregulated in α-CD3/α-CD28 stimulated naïve CD4^+^ T cells compared to control cells (Fig. 3C, Table S1). Consequently, we asked whether the Rb/E2f axis, essential for the G1 to S-phase transition, is differentially regulated in PHB2-deficient T cells. While HELLS protein was not detected in both unstimulated control and PHB2-deficient T cells, stimulation with α-CD3/α-CD28 revealed a robust upregulation of HELLS in control T cells. In contrast, naïve CD4^+^ T cells isolated from *Phb2*^*TKO*^ were not able to upregulate HELLS expression after stimulation, indicating a role for PHB2 in the regulation of HELLS proteostasis (Fig. 4F). Additionally, Western blot analysis revealed a lower phosphorylation of Rb protein in TCR-stimulated PHB2-deficient naïve CD4^+^ T cells compared to control T cells (Fig. 4F).

Another possible explanation for the decreased T cell numbers in *Phb2*^*TKO*^ mice could be an increased sensitivity to apoptosis, leading to the demise of cells attempting to enter the S-phase of the cell cycle^22^. However, Annexin V staining of PHB2-deficient T cells stimulated for two days with α-CD3/α-CD28 revealed no difference in apoptotic cells, pre-apoptotic cells, or live cells compared to controls (Fig. 4G, Supplementary Fig. 4D). Surprisingly, dead cells were significantly reduced in PHB2-deficient T cells compared to control cells (Fig. 4F).

In summary, PHB2-deficient T cells are unable to proliferate and subsequently differentiate into effector T cell subsets. Apoptosis as an explanation for reduced cell count and proliferation can be excluded since PHB2-deficient T cells did not exhibit increased sensitivity to apoptosis after stimulation, which is in contrast to other PHB2-deficient cell types reported previously^1,3^. Their failure to enter the S-phase of the cell cycle during proliferation and a reduction in RB phosphorylation suggest a general block of cell cycle progression at the G1- to S-phase transition, thus blocking proliferation and differentiation of activated T cells.

### PHB2 deficiency is indispensable for T cell activation

**Fig. 5:**
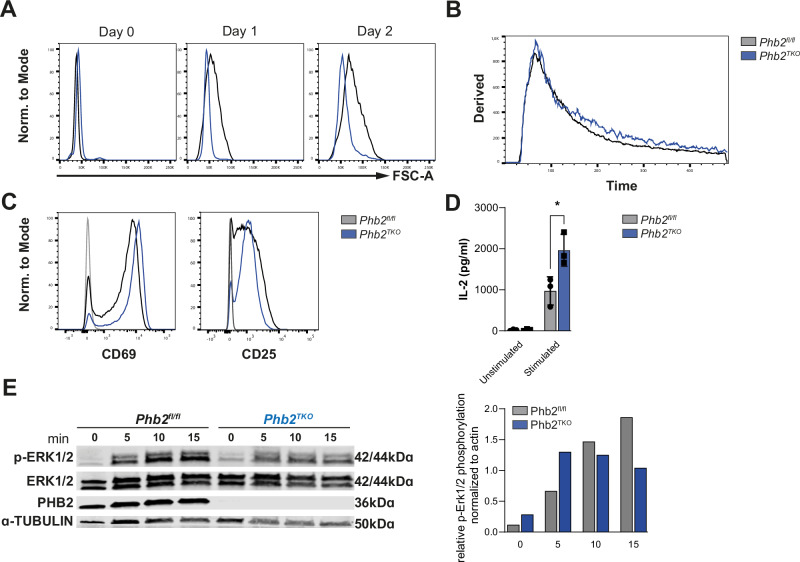
PHB2 deficiency does not influence the activation of T cells. **A** Representative histograms of forward scatter area (FSC-A) for cell size of in vitro α-CD3/α-CD28 stimulated naïve CD4^+^ T cells at 0, 1, and 2 days post-stimulation with *n* = 4/genotype (**B**) Representative histogram of calcium flux in naïve CD4^+^ T cells following activation by biotin-α-CD3/CD4 cross-linkage (**C**) Representative histogram of CD69 and CD25 expression on naïve CD4^+^ T cells after 24 h of activation with α-CD3/α-CD28, with *n* = 4/genotype. **D** IL-2 secretion by naïve CD4^+^ T cells after 24 h of in vitro activation with α-CD3/α-CD28, measured by ELISA, with *n* = 3 mice/genotype **E** Western blot analysis and normalization of Erk1/2 phosphorylation in CD4^+^ naïve T cells after in vitro activation with α-CD3/α-CD28, using pooled samples from 4 mice per genotype The data presented are representative of at least three independent (**A**–**D**) and two (**E**) experiments, with similar results. Analyses were performed on 8–12-week-old mice. Bar graphs show means +/- SDs and single values (**D**). Statistical significance was calculated using an unpaired two-tailed t-test with Holm-Šidák correction for multiple comparisons. *p < 0.05.

T cell activation, proliferation, and differentiation into effector and memory T cells involve massive remodeling of T cell size, molecular content, and create a massive increase in the demand for energy and amino acids^23^. In general, activation of T cells leads to an increase in cell size, also referred to as blasting^24^. The proliferative defect of PHB2-deficient T cells in response to TCR stimulation and T helper cell subset cytokine cocktails suggests possible alterations downstream of TCR signaling and its co-stimulatory receptor CD28. To test whether PHB2-deficient T cells can blast after TCR activation, MACS-purified naive CD4^+^ T cells were either left untreated or cultured for one or two days in the presence of α-CD3/α-CD28stimulation. FACS analysis revealed that PHB2-deficient T cells did not increase their size one day after stimulation and were less efficient in increasing their cell size compared to control T cells after two days of stimulation (Fig. 5A).

The primary activation of T cells is mediated by the binding of the TCR to the MHC II molecule of antigen-presenting cells. This receptor-ligand interaction leads to a sudden and very fast rush of Ca^2+^ into the cytoplasm mediated by calcium channels in the plasma membrane and the endoplasmic reticulum, and subsequently promotes T cell proliferation^25^. We measured calcium flux by flow cytometry with the calcium-sensitive dyes Fluo-4 and FuraRed. Calcium flux of PHB2-deficient naïve CD4^+^ T cells upon CD3 and CD4 cross-linkage revealed no significant difference in calcium influx compared to control T cells (Fig. 5B). Prolonged activation of T cells then leads to the upregulation of activation markers such as CD69 and CD25^26,27^. PHB2-deficient T cells showed no differences in CD25 expression 24 h after α-CD28/α-CD28 TCR stimulation compared to control T cells, while the expression of CD69 was even increased (Fig. 5C). Together, this suggests that PHB2-deficient T cells retain functional signaling downstream of the TCR. The secondary activation through CD28 additionally induces the production and secretion of IL-2, a survival and pro-proliferative cytokine^28,29^, which in turn can potentiate itself in a paracrine manner through IL-2 signaling via its receptor CD25^28^. We measured IL-2 secretion of naive PHB2 and control CD4^+^ T cells after 24 h of α-CD28/α-CD28 stimulation by ELISA. Surprisingly, PHB2-deficient T cells produced significantly more IL-2 than control T cells (Fig. 5D).

Buehler et al. recently reported that plasma-membrane localized prohibitins affect Erk1/2 signaling, thereby altering Th17 differentiation. To analyze Erk1/2 signaling of PHB2-deficient T cells, we stimulated naïve CD4^+^ T cells with α-CD3/α-CD28 for 0, 5, 10, and 15 min. After 5 min of stimulation, Erk1/2 phosphorylation was successfully upregulated in PHB2-deficient T cells similar to control T cells, but declined at later timepoints while, as expected, the phosphorylation of control T cells further increased (Fig. 5E). However, as reported by Buehler et al., the inhibition of prohibitins on the plasma membrane, which solely reduces Erk1/2 signaling, did not lead to decreased proliferation. Therefore, our data does not suggest that this reduction in signaling alone can account for the lack of proliferation of PHB2-deficient T cells^10^.

These results, combined with the proteomics analysis, suggest that PHB2-deficient T cells are capable of receiving their primary and secondary signals through the TCR and CD28, respectively.

### The mitochondrial structure and function of T cells are dependent on the presence of PHB2

**Fig. 6:**
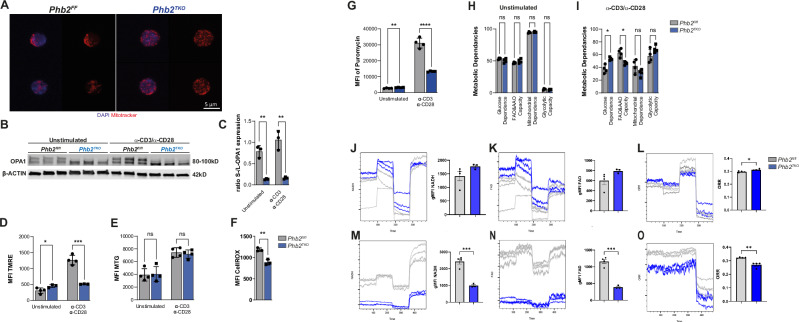
PHB2 is essential for mitochondrial structure and function. **A** Representative immunohistochemical staining of MACS CD4^+^ T cells, co-stained with DAPI and Mitotracker Orange CMTMRos **B** Western Blot analysis of OPA1 expression in naïve CD4^+^ T cells, comparing unstimulated cells (left), stimulated with α-CD28/α-CD28 (right) for 24 h, with *n* = 3 mice/genotype (**C**) Statistical analysis of the relative S-/L-OPA1 ratio from B. (*n* = 3 mice/genotype) (**D**) The ΔΨm (TMRE) of naïve CD4^+^ T cells, either unstimulated or stimulated for 24 h with α-CD28/α-CD28. (*n* = 3-4 mice/genotype) (**E**) Mitochondrial mass (MTG) of naïve CD4^+^ T cells unstimulated or stimulated for 24 h with α-CD28/α-CD28 (*n* = 4) (**F**) Bar graph depicting the levels of reactive oxygen species (ROS) in CD4^+^ naïve T cells after 24 h of stimulation with α-CD28/α-CD28, with *n* = 3 mice/genotype (**G**) Single-cell ENergetIc metabolism by profiling Translation inHibition (SCENITH) analysis of CD4^+^ naïve T cells, unstimulated or stimulated for 24 h with α-CD28/α-CD28, with pre-gating for CD25 expression in the stimulated cells. (*n* = 4 mice/genotype) Metabolic profile of unstimulated (**H**) and stimulated (**I**) CD4^+^ naïve T cells from G. Stimulated cells were pre-gated for CD25 (*n* = 4) (**J**–**O**) Redox balance analysis by autofluorescence measurement of NADH and FAD in CD4⁺ T cells from Phb2^fl/fl^ and *Phb2*^*TKO*^ mice at steady state (**J–L**) and following αCD3/αCD28 activation (**M–O**). (**J**, **M**) Geometric mean fluorescence intensity (gMFI) of NADH levels. (**B**, **E**) Geometric mean fluorescence intensity (gMFI) of FAD levels. (**L**, **O**) Optical redox ratio kinetics. Barplots depict gMFI levels of NADH and FAD at baseline, before the addition of any inhibitors. Data is representative of at least three (**D**, **E**, **G**–**I**), two (**B**, **C**), and one (**A**, **F**) independent experiments. Analyses were performed on 8-12-week-old mice. Bar graphs show means +/- SDs and single values (**C**–**I**). Statistical significance was calculated using an unpaired two-tailed t-test with Holm-Šidák correction for multiple comparisons. *p < 0.05, **p ≤ 0.01, ***p ≤ 0.001, ****p ≤ 0.0001.

Multiple studies have reported that the mitochondrial structure is disrupted in cells lacking PHB2^1,3,4,30^. To assess the mitochondrial structure in PHB2-deficient T cells, we stained CD4^+^ T cells with Mitotracker Orange, a mitochondrial dye that is independent of the mitochondrial membrane potential. We found that, while control T cells possessed long concentrated tubular mitochondrial bodies, mitochondria of PHB2-deficient T cells were distributed in smaller bodies throughout the cell (Fig. 6A), displaying a fissioned phenotype, a process where mitochondria divide or segregate into two separate mitochondrial organelles. It was previously reported that the disruption of the homeostasis of the mitochondrial structure in PHB2-deficient cells is due to a disbalanced ratio of the two isoforms L-OPA1/S-OPA1^1^. Indeed, Western blot analysis of L-OPA1 of untreated as well as α-CD28/α-CD28 stimulated T cells showed a distorted ratio of S-OPA1/L-OPA1 in naïve CD4^+^ T cells isolated from *Phb2*^*TKO*^ mice (Fig. 6B, C). To evaluate if this ultimately leads to dysfunctional mitochondria, we stained the mitochondrial membrane potential (ΔΨm) at steady state and after TCR stimulation with the potential-dependent mitochondrial membrane dye TMRE. This analysis revealed a small but significant increase in membrane potential in PHB2-deficient T cells at steady state compared to control T cells (Fig. 6D). After stimulation, however, PHB2-deficient T cells did not increase their membrane potential compared to control T cells (Fig. 6D). Since T cells can increase their mitochondrial volume during activation up to fourfold^31^ and this could explain the difference in mitochondrial membrane potential after activation, we stained unstimulated and stimulated T cells with Mitotracker Green, a membrane potential independent mitochondrial dye, to investigate stimulation induced mitogenesis. Surprisingly, PHB2-deficient naïve CD4^+^ T cells displayed a similar increase in mitochondrial volume 24 h after TCR stimulation compared to wild-type cells. (Fig. 6E). A dysfunctional membrane potential can be an indicator of a deficiency in the electron transport chain. Staining with CellROX, a ROS-dependent dye, showed a reduced amount of ROS produced by PHB2-deficient T cells compared to control mice (Fig. 6F). Together, these findings suggest that PHB2-deficient T cells cannot increase ATP production upon activation since the ΔΨm directly drives the conversion of ADP to ATP by complex V of the ETC^32^.

To investigate if the reduced mitochondrial membrane potential seen after TCR stimulation translates into alterations in T cell metabolism, activated T cells were incubated with puromycin, an antibiotic that is incorporated into newly synthesized peptide chains at the ribosome during translation^33^. Since protein translation consumes up to 50% of the cell’s energy, any difference in energy generation also impacts the de novo synthesis of proteins^34^. Analysis of activated T cells revealed that the expression of CD25 and puromycin incorporation correlated positively (Supplementary Fig. 4A); thus, we pre-gated on CD25 on stimulated T cells. At steady state, PHB2-deficient T cells showed a small but significant increase in de novo protein synthesis. Interestingly, activated PHB2-deficient T cells upregulated protein translation after 24 h of in vitro stimulation; however, they only upregulated protein synthesis to about half of that of control cells (Fig. 6G, Supplementary Fig. 4B, C).

Next, naïve CD4^+^ T cells were treated with different metabolic inhibitors to analyze their metabolic profile by SCENITH^35^. Oligomycin was used to block mitochondrial-derived ATP, and 2-DG to block glycolytic-derived ATP. The relative amount of protein synthesis that a cell can still conduct after treatment with one of these inhibitors indicates how dependent a cell is solely on glycolysis or mitochondrial ATP generation (Supplementary Fig. 4B, C). SCENITH analysis of naïve PHB2-deficient CD4^+^ T cells showed no differences compared to control cells (Fig. 6H), consistent with the fact that resting T cells mainly rely on mitochondrial respiration as their ATP source^36^. However, after α-CD28/α-CD28 stimulation, PHB2-deficient T cells showed a higher dependence on glycolysis and a subsequent reduced capacity for fatty acid (FAO) and amino acid oxidation (AAO) (Fig. 6I).

To gain a deeper understanding of how PHB2 affects mitochondrial dynamics, we performed a redox balance analysis to measure NADH and FAD levels, which serve as indicators of mitochondrial activity. At steady state, no statistically significant differences in NADH or FAD levels were detected, suggesting that the mitochondria are functioning normally (Fig. 6J–L). However, upon TCR activation with α-CD3/α-CD28, PHB2-deficient CD4⁺ T cells failed to increase NADH or FAD levels compared to wild-type control cells, which are indicators of mitochondrial activity (Fig. 6M–O). The optical redox ratio, which reflects mitochondrial activity per mitochondrial unit, was decreased in PHB2-deficient T cells, suggesting the inability to mobilize the Krebs (TCA) cycle. This phenotype aligns with the lower TMRE levels observed, suggesting defects in mitochondrial OXPHOS-driven respiration. PHB2-deficiency seems to disrupt mitochondrial dynamics by preventing proper spatial coordination of the complexes in the electron transport chain. Although T cell activation still induced mitochondrial biogenesis, as observed with MitoTracker staining, the inability of PHB2-deficient T cells to generate functional mitochondria upon activation likely underlies the reduced protein synthesis capacity measured by SCENITH, considering that protein synthesis is an ATP-enabled biological process.

Together, these data suggest that PHB2-deficient T cells are unable to increase metabolic activity to meet the metabolic and anabolic demand after stimulation, which may render them unable to proliferate.

## Discussion

Previous studies on PHB2 in T cells investigated T cell lines or targeted PHB2 specifically at the plasma membrane^10,37^. By utilizing the CD4-cre mouse line, we were able to investigate the role of PHB2 selectively in T cells in vivo. Analysis of these mice revealed a crucial role for PHB2 in the maintenance, differentiation, and activation of T cells. T cells were greatly reduced at steady state and unable to mount an effective immune response after immunization with MOG peptide. Furthermore, the genetic deletion of *PHB2* specifically in Treg cells led to a scurvy-like phenotype, demonstrating an essential role for PHB2 in Treg cells.

We found that although the thymic development was unaffected, all peripheral T cell populations were significantly reduced at steady state in spleen, LN, and mLN in *Phb2*^*TKO*^ mice. Interestingly, PHB2 deficiency did not manifest in the thymus of *Phb2*^*TKO*^ mice, even though CD4-cre deletion is already active at the double positive stage during T cell development, but was fully established in peripheral naïve T cells. One possible explanation could be that the PHB2 protein has a long half-life and that cre-mediated deletion only manifests in the periphery.

Some peripheral effector T cells still expressed PHB2, possibly due to the negative selection pressure and/or incomplete recombination by the cre. The fact that only PHB2-expressing T cells proliferated in vitro underscored this selection pressure imposed by PHB2 deficiency.

Previous studies on PHB2 in cell lines and mouse models already suggested that PHB2 has a conserved important role for different cell functions, such as proliferation and apoptosis^38–40^. We observed that PHB2-deficient T cells were incapable of proliferating. A proliferative defect has previously been reported in other PHB2-deficient tissues and cells^1,3,4^, and could be partially explained by the loss of L-OPA1, but it has not been specifically documented in T cells. These proliferation deficiencies were associated with increased sensitivity towards apoptosis in PHB2-deficient cells^1,41,42^. In one study on β-cells, the authors found that increased proliferation was accompanied by an increase in apoptosis^3^.

In contrast to other cell types, we did not detect an increased rate of apoptosis in stimulated PHB2-deficient T cells. Instead, cell cycle analysis revealed that the lack of proliferation may be due to a defect in entering the S phase and synthesizing DNA. This is further supported by the fact that we found S-phase-associated proteins in our proteomics data set, one of them being HELLS to be significantly downregulated. HELLS is a helicase that establishes DNA methylation patterns^43^ and is essential for mature T cell proliferation^18^, thus representing one of the possible causes why PHB2-deficient T cells cannot proliferate.

In order to exclude the possibility that defective T cell receptor (TCR) activation is the cause of the proliferation defect, we performed calcium flux experiments upon TCR stimulation. Our results showed that PHB2-deficient T cells can initiate calcium flux. After 24 h of stimulation, these cells also express the typical activation markers, such as CD25 and CD69. This suggests that signaling downstream of the TCR is intact, as CD69 expression is regulated by NF-κB, ERG-1, and AP-1^26^, while CD25 expression is regulated by NFAT and AP-1^44^.

Unexpectedly, PHB2-deficient T cells produced higher levels of IL-2 in vitro than control T cells. IL-2 is induced by downstream TCR transcription factors (NFAT, AP-1, and NF-κB). This finding contradicts previous reports, which linked PHB2 to reduced IL-2 production in T cells. However, these earlier studies focused on surface-bound PHB2. This discrepancy suggests that surface-bound and mitochondrial PHB2 perform distinct functions in T cells^45–47^. Another study showed that targeting surface-localized prohibitins in T cells reduced MAPK-induced signaling, which reduced Th17 induction and promoted Treg cell induction, without affecting CD4 T cell proliferation^10^. Contrary to this, PHB2-deficient murine T cells exhibited a strong proliferation deficiency in our study. Overall, this rather unexpected finding can be attributed to altered regulation of IL-2 gene expression. PHB2 is known to interact with the transcription factor NF-κB, which plays a crucial role in the regulation of IL-2 gene expression. In the absence of PHB2, the NF-κB pathway may be overactivated, leading to increased IL-2 production. Alternatively, PHB2 may be involved in the negative regulation of IL-2 production, and its deficiency may result in the loss of this inhibitory effect, leading to increased IL-2 production.

A lack of PHB2 is associated with a fragmented mitochondrial phenotype, as documented in previous studies^1,16^. This effect is directly linked to an imbalanced ratio between L-OPA1 and S-OPA1, resulting from the dysregulated processing of L-OPA1. In our study, we discovered that PHB2-deficient T cells also exhibit an imbalanced L-OPA1/S-OPA1 ratio and fragmented mitochondrial structure. Surprisingly, our proteomics data showed that the i-AAA protease YME1L1 is downregulated in activated T cells lacking PHB2. It has been previously demonstrated that YME1L regulates the processing of L-OPA1 through OMA1, and its absence leads to mitochondrial fragmentation^16,48^. Furthermore, it was shown that it supports cell proliferation^48^. This may contribute to the defect in T cell proliferation; however, it does not account for the fragmentation of mitochondria at steady state.

Mitochondria of PHB2-deficient T cells displayed a normal membrane potential (ΔΨm) at steady state, but these cells failed to upregulate the ΔΨm upon TCR activation. This suggests a metabolic dysfunction, as the ΔΨm is crucial for the generation of ATP at complex V of the electron transport chain^32^. A subsequent analysis using the flow cytometry-based method to functionally profile metabolism with single-cell resolution (SCENITH) revealed that T cells lacking PHB2 are incapable of enhancing their metabolic activity after activation. This data is underlined by the redox measurement of NADH and FAD electron carrier molecules. Specifically, we noticed a significant increase in glucose dependence and a decrease in fatty acid and amino acid oxidation of PHB2-deficient T cells compared to control cells. The inability to increase metabolic activity after activation implies that the proliferative defect of PHB2-deficient T cells may be due to their inability to support proliferation both at the metabolic and anabolic levels.

Surprisingly, our IPA analysis of stimulated PHB2-deficient T cells revealed no differentially regulated pathways related to T cell function or proliferation. However, the tRNA charging pathway was significantly upregulated in both unstimulated and stimulated PHB2-deficient T cells. This pathway involves aminoacyl-tRNA synthetases (aaRSs), enzymes that catalyze the esterification of amino acids to their corresponding tRNAs, a crucial step in the translation of proteins. There are two types of aaRSs: those in the mitochondria and those in the cytoplasm^17^. There was no bias between mitochondrial and cytoplasmic aaRS variants in our data, suggesting no preferential regulation of either type. The absence of a known master regulator of tRNA synthetases in T cells suggests a possible new direction for the function of PHB2.

In conclusion, this study shows that PHB2 is crucial for T cell homeostasis, proliferation, and effector function and possesses distinct functions in relation to its cellular location. Our findings show that PHB2 is indispensable for the proliferation and differentiation of T cells, with PHB2-deficient T cells exhibiting a significant cell cycle arrest during the transition from G1 to S phase. Moreover, PHB2-deficient T cells fail to upregulate their metabolism sufficiently after activation, resulting in diminished protein synthesis. These findings emphasize the importance of PHB2 in maintaining the integrity of T cell responses. Future research should investigate the molecular mechanisms underlying PHB2’s role in metabolic regulation and cell cycle progression, as well as its potential as a therapeutic target for immune-related disorders.

## Methods

### Mice

*Phb2*^*fl/fl*^ mice were crossed to *CD4Cre* mice to obtain *Phb2*^*TKO*^ mice (B6-Phb2tm1Tlan Tg(Cd4-cre)1Cwi/Tarc). *Phb2*^*fl/fl*^ mice were crossed to *Foxp3Cre* mice to obtain *Phb2*^*TregKo*^ mice (B6-Phb2tm1Tlan Foxp3tm1(cre)Saka/Tarc). Mice were of the C57BL/6 J strain, 8–12-week-old, unless otherwise stated, sex-matched male and female genders, and housed under specific pathogen-free conditions. All experiments were in accordance with the guidelines of the Translational Animal Research Center (TARC) of the University of Mainz and approved by the institutional committee on animal experimentation and the government of Rheinland-Pfalz (RLP). We have complied with all relevant ethical regulations for animal use. On the day of the experiment, animals were euthanized by gradual-fill CO₂ inhalation in accordance with institutional and national guidelines. The flow rate was set to displace approximately 30% of the chamber volume per minute. Death was confirmed by cervical dislocation.

### Single cell preparation

Single cell suspensions of thymus, spleen, lymph nodes, and mesenteric lymph nodes were prepared by smashing organs through a 40 µm filter in PBS supplemented with 2% fetal calf serum (FCS). Following, red blood cell lysis was conducted on spleenocytes.

### Antibodies and flow cytometry

Single cell suspension was used for flow cytometric stainings. First, Fc-block (5 µg/mL) was used for 15 min at 4 °C. Surface markers were then stained for 20 min at 4 °C with antibodies against TCRb-Fitc (Biolegend #109205), TCRb-PE-Cy7 (Biolegend #109221), CD19-PE-Cy7 (Biolegend #115520), CD4-PerCP (Biolegend #100432), CD4-BV510 (Biolegend #100559), CD4-PE (Biolegend #100408), CD8-Pacifique Blue (Biolegend #100725), CD8-BV510 (Biolegend #100752), CD62L-APC (Biolegend #104412), CD44-PE (eBioscience #12-0441), CD44-Fitc (eBioscience #11-0441), CD25-Fitc (BD Biosciences #553072), CD45.1-PE-Cy7 (Biolegend #110730), CD45.2-Fitc (eBioscience #11-0454), FVD-APC-eFl780 (eBioscience #65-0865), FVD-eFl506 (eBioscience #65-0866) and CD69-Fitc (eBioscience #11-0691). After staining, the cells were fixed in 2% PFA for 15 min at room temperature. For intracellular staining, cells were stained for Ki67-APC (Biolegend #652405) and Foxp3-APC (eBioscience 17-5773) with the Bioscience™ Foxp3/Transcription Factor Staining Buffer Set (00-5523-00).

Cells were acquired with a FACSCanto II cytometer (BD Bioscience) using FACS Diva software (BD Bioscience). Flow cytometry data were analyzed with FlowJo software version 10 (BD Bioscience). For all analyses, doublets (FSC and SSC properties) and dead cells (dye inclusion, fixable viability dye APC-ef780 eBioscience) were excluded.

### EAE Induction

Active EAE immunization with MOG_35–55_ (GenScript) peptide emulsified in complete Freund´s adjuvant (CFA) (Difco), along with pertussis toxin (List Biological Laboratories) injections and disease assessment was performed as described elsewhere (REF). Brief, 50 μg MOG35–55/CFA was injected subcutaneously into the tail base of the mouse. On the day of immunization and 2 days later, 150 ng pertussis toxin in PBS was applied i.p. unless otherwise stated.

### MACS purification and in vitro stimulation

CD4^+^ naïve T cells were purified using the naïve CD4^+^ T cell Isolation kit for mouse from Miltenyi Biotec (130-104-453). For short-term kinetics, 10 × 10^6^/ml naïve CD4^+^ T cells were stimulated with 1 µg/ml of α-CD3 and α-CD28 for the indicated time points. For 24 h stimulations, 200 000/well naïve CD4^+^ T cells were stimulated in a 96- well plate coated with 1 µg/ml α-CD3, and added 1 µg/ml α-CD28 to the medium.

For in vitro proliferation assay, cells were labeled with CellTrace violet cell proliferation kit (VCT) according to the manufacturer (Invitrogen, C34557). For in vitro polarization, all cells were stimulated in a 96- well plate coated with 1 µg/ml α-CD3 and added with 1 µg/ml α-CD28 to the media. Additionally, 2 ng/ml TGF-β, 25 ng/ml IL-6, and 10 µg/ml α-IFNγ for Th17 and 2 ng/ml TGF-β, 10 µg/ml α-IFNγ, and 20 ng/ml IL-2 for Treg polarization were added.

### Membrane potential and Mitotracker green staining

Stimulated naïve CD4^+^ T cells were incubated with 25 nM/ml TMRE (Abcam #AB113852) for membrane potential and 100 nM Mitotracker Green FM (Thermo Fischer Scientific M7514) for mitochondrial mass for 40 min at 37 °C and 5%CO_2_. Following cells were stained for surface markers as described above.

### Mitotracker red staining

Cells were prepared at a concentration of 10 × 10^6^/mL in 500 µL RPMI-1640 medium supplemented with heat-inactivated 10% FCS. After washing with RPMI-1640, 500 µL of MitoTracker Orange CMTMRos (M7510, Thermo Scientific, 1 µM) was added, and cells were incubated at 37 °C for 15 min. After washing the cells with 0.5% BSA/PBS, 500 µL of 4% PFA in PBS containing 0.2 µg/mL Hoechst 33342 was added, and cells were incubated at room temperature for 10 min. Cells were washed twice with 0.5% BSA/PBS, resuspended in 50 µL of Mowiol 4-88 mounting medium, and mounted on glass slides with coverslips. The slides were incubated overnight before microscopy analysis.

### Cell cycle analysis

For cell cycle analysis, cells were fixed with the eBioscience™ Foxp3/Transcription Factor Staining Buffer Set (00-5523-00). Next, cells were incubated with RNAse (100 µg(ml) for 15 min at 37 °C. Propidium Iodide (50 µg/ml, P4170 Sigma Aldrich) was added right before acquisition to the cells.

### Calcium flux

For calcium flux, cells were stained with Fluo-4 (Thermo Fisher Scientific 1:250) and FuraRed (Thermo Fisher Scientific 1:100). For calcium flux induction, cells were stained with 5 µg/ml α-CD3 biotin (100304 Biolegend) and 5 µg/ml α-CD4 biotin (13-0041 eBioscience). For cross connection, 1 mg/ml Neutravidin was added right before acquisition (Thermo Fischer Scientific 31000).

### SCENITH staining

For metabolic profiling, stimulated naïve CD4^+^ T cells were incubated with metabolic inhibitors for 25 min. 2 µM Oligomycin A (Sigma Aldrich 75351), 100 mM 2DG (Sigma Aldrich D8375). Then, 10 µg/ml Puromycin (Hycultec) was added for 40 Minutes. Cells were first stained for surface makers, then stained with the anti-puromycin antibody (EMD Millipore MABE343-AF647) with the eBioscience™ Foxp3/Transcription Factor Staining Buffer Set (00-5523-00).

### Autofluorescence-based redox balance analysis

Splenocytes from Phbt2^fl/fl^ and Phbt2^TKO^ were plated at around 5 × 10^6^ cells/mL and activated overnight (16–20 h) with soluble anti-CD3 and anti-CD28 in RPMI complete medium, following the established procedure. After activation, cells were washed and resuspended in 500 μL glucose-only DMEM without phenol red (Gibco) for autofluorescence deconvolution analysis, using a FACSymphony A5 SE (BD Biosciences). Samples were left at room temperature to stabilize metabolic activity. The Seahorse-mitostress test was adapted, as previously described (Abir et al., 2024). Briefly, metabolic inhibitors were sequentially added during acquisition, with 1 min incubations at room temperature between each step: Baseline at steady-state, followed by oligomycin (20 µM), FCCP (10 µM), Rotenone/Antimycin A (2 µM each). Cells were immediately returned to the cytometer for continued acquisition. NAD(P)H and FAD signals were detected in the UV region of the spectrum. For NAD(P)H, emission was detected at 450/50 nm, while for FAD, emission was detected at 585/30 nm. The optical redox ratio was calculated as FAD / (NAD(P)H + FAD). Dead cells were excluded via staining with 7-AAD dye (1:200 dilution.ratio).

### Apoptosis assay

Stimulated naïve CD4^+^ T cells were washed once with Annexin V staining buffer and then stained with Annexin V (Immunotools 31490016) antibody and propidium Iodide (PI) in Annexin V staining buffer for 20 minutes at 4 °C. Annexin V staining Buffer 10x recipe: 0.1 M HEPES, 1.4 M NaCl, 25 mM CaCl_2_.

Freshly isolated splenocytes from *Phb2*^*fl/fl*^ and *Phb2*^*TKO*^ mice were MACS-sorted for CD90^+^ T cells isolation. 1 ×10^6^ cells were cultured overnight at 37 °C in 5% CO₂ in RPMI-1640 complete medium (10% FBS, 1% penicillin/streptomycin, 2 mM L-glutamine, 50 µM β-mercaptoethanol) under the following conditions: Unstimulated (resting), activated with a-CD3 (1 µg/mL), or with a-CD3 (1 µg/mL) and a-CD28 (1 µg/mL). After overnight incubation, cells were washed with cold PBS and stained with Annexin V-APC (Immunoblots 31490016) and propidium iodide (PI). Staining was performed in Annexin V Binding Buffer (0.01 M HEPES, 0.14 M NaCl, 2.5 mM CaCl_2_). Briefly, 100.000 T cells were resuspended in 200 µL of binding buffer, incubated with Annexin V-APC and PI for 15 min at room temperature in the dark. Samples were acquired immediately on a FACS Canto II flow cytometer and analyzed using FlowJo v10, by gating on the CD90^+^CD4^+^ T cell population. Viable (Annexin V⁻ PI⁻), early apoptotic (Annexin V⁺ PI⁻), late apoptotic/necrotic (Annexin V⁺ PI⁺), and free nuclei (Annexin V^-^PI^+^) populations were quantified.

### Western blot analysis

Cells were lysed in RIPA buffer (Cell Signaling Technology 9806) at 10 × 10^6^ cells per 50 µl. PhosSTOP (Roche 04906837001) and protease inhibitor (Roche 04693132001) were added to the lysis buffer as per the manufacturer’s instructions. 10 µg protein per sample was separated by gel electrophoresis on TGX Stain-Free Precast Gels (BioRad 4568083). Primary Antibodies: PHB1 (1:1000; Cell Signaling #2426) PHB2 (1:1000; Biolegend #611801), b-actin (1:1000; Signaling #4970), HELLS (1:1000; Cell Signaling #7998), Erk1/2 (1:1000; Cell Signaling #9102), p-Erk1/2 (1:2000; Cell Signaling #9106), a-tubulin (1:1000; Cell Signaling #2144), OPA1 (1:500; BD #612606), p-Rb (1:2000; Cell Signaling #9309). Secondary Antibodies: Anti-rabbit IgG DyLight 800 4X PEG Conjugate (1:30000; Cell Signaling #5151), Anti-rabbit IgG DyLight 680 Conjugate (1:15000; Cell Signaling #5366). Western Blots were acquired on the Li-Cor Odyssey imaging system and analyzed using Image Studio Lite 5.2.1.

### Preparation of enriched mitochondrial fractions from T cells

Between 30–60 million T cells were collected and washed with cold PBS, followed by centrifugation at 400 *g* for 5 min. The cells were resuspended in isolation buffer (250 mM sucrose, 10 mM HEPES pH 7.4, 1 mM EDTA, and protease inhibitors) and homogenized using a Dounce homogenizer (15–20 strokes). Homogenates were centrifuged at 800 *g* for 10 min at 4 °C to collect the nuclear fractions. The supernatants were transferred to new tubes and centrifuged at 10,000 *g* for 10 min at 4 °C. The mitochondrial pellet was resuspended in isolation buffer. Protein concentrations were assessed using the BCA assay. The enrichment of the fractions was tested by Western blot using the following antibodies: actin (1:1000, Proteintech 20536-1-AP) as a cytosolic control; VDAC1 (1:1000, Neuromab N152B/23) and Tom20 (1:500, Sigma HPA011562) as mitochondrial markers; and PHB2 (1:1000, Biolegend #611801).

### IL-2 ELISA

IL-2 in supernatant was measured with the BD OptEIA^TM^ Mouse IL-2 ELISA Set (BD Bioscience #555148). In brief, microwells were coated with 100 µL per well of capture antibody diluted in coating buffer, using the dilution recommended in the lot-specific instruction/analysis certificate. Plates were sealed and incubated overnight at 4 °C. Wells were then aspirated and washed three times with at least 300 µL wash buffer per well, followed by inversion and blotting on absorbent paper to remove residual liquid. Plates were blocked with at least 200 µL assay diluent per well and incubated for 1 h at room temperature. After blocking, wells were aspirated and washed as described above. Standards, samples, and controls were prepared in assay diluent, and 100 µL of each was added to the appropriate wells. Plates were sealed and incubated for 2 h at room temperature. Wells were then aspirated and washed five times. Subsequently, 100 µL of working detector solution (detection antibody combined with SAv-HRP reagent) was added to each well, and plates were incubated for 1 h at room temperature. After incubation, wells were aspirated and washed seven times, including a soaking step of 30 s to 1 min during each wash. Following the final wash, 100 µL of substrate solution was added to each well, and the plate was incubated for 30 min at room temperature in the dark without a plate sealer. The reaction was stopped by adding 50 µL of stop solution to each well. Absorbance was measured at 450 nm within 30 min, with wavelength correction performed at 570 nm when available. ELISA was acquired on the TECAN plate reader.

### Mass spectrometry

#### Proteomics sample preparation

Cells were lysed in 100 µL urea-containing buffer (7 M Urea, 2 M Thiourea, 1% Sigma Aldrich Phosphatase Inhibitor Cocktail 3 in 100 mM NH_4_HCO_3_) by sonication for 15 min (30 s on/off cycles) at 4 °C with high power in a Bioruptor device (Diagenode, Liège, Belgium). Subsequently, proteins were digested using filter-aided sample preparation (FASP) as previously described in detail^49^. In brief, lysates were loaded onto spin filter columns (Nanosep centrifugal devices with Omega membrane, 30 kDa MWCO; Pall, Port Washington, NY) and washed three times with buffer containing 8 M urea. Afterwards, proteins were reduced and alkylated using DTT and iodoacetamide (IAA), respectively. After alkylation, excess IAA was quenched by the addition of DTT. Buffer was then exchanged washing the membrane three times with 50 mM NH_4_HCO_3_ and proteins digested overnight at 37 °C using trypsin (Trypsin Gold, Promega, Madison, WI) at an enzyme-to-protein ratio of 1:50 (w/w). After proteolytic digestion, peptides were recovered by centrifugation and two additional washes with 50 mM NH_4_HCO_3_. After combining peptide flow-throughs, samples were acidified with trifluoroacetic acid (TFA). A peptide aliquot corresponding to 5 µg of protein was lyophilized and reconstituted in 25 µL 0.1% formic acid (FA) for whole proteome analysis.

#### Liquid chromatography mass spectrometry (LC-MS)

For the LC-MS analysis of the full proteome, 100 ng of the reconstituted peptides were separated on a nanoElute LC system (Bruker Corporation, Billerica, MA, USA) at 400 nL/min using a reversed-phase C18 column (Aurora UHPLC emitter column, 25 cm × 75 µm 1.6 µm, IonOpticks) which was heated to 50 °C. Peptides were loaded onto the column in direct injection mode at 600 bar. Mobile phase A was 0.1% FA (v/v) in water, and mobile phase B 0.1% FA (v/v) in ACN. Peptides were separated by running a linear gradient from 2% to 37% mobile phase B over 39 min. Afterwards, the column was rinsed for 5 min at 95% B. Eluting peptides were analyzed in positive mode ESI-MS using parallel accumulation serial fragmentation (PASEF) enhanced data-independent acquisition mode (DIA) on a timsTOF Pro 2 mass spectrometer (Bruker Corporation)^50^. The dual TIMS (trapped ion mobility spectrometer) was operated at a fixed duty cycle close to 100% using equal accumulation and ramp times of 100 ms each, spanning a mobility range from 1/K_0_  =  0.6 Vs cm^−2^ to 1.6 Vs cm^−2^. We defined 36 × 25 Th isolation windows from *m/z* 300 to 1,165, resulting in fifteen diaPASEF scans per acquisition cycle. The collision energy was ramped linearly as a function of the mobility from 59 eV at 1/K_0_  =  1.3 Vs cm^−2^ to 20 eV at 1/K_0_  =  0.85 Vs cm^−2^. All samples were measured in triplicate.

#### Raw data processing

Peptides were identified, and label-free quantification (LFQ) of proteins was performed using DIA-NN (v1.8)^51^. Full proteome samples were processed using library-free mode with standard parameters, except for tryptic cleavage sites, considering no cleavage before proline. The FASTA protein database contained 17085 reviewed (Swissprot) protein entries of the mouse reference proteome and 172 common contaminant proteins and was obtained on 17.02.2022 from uniprot.org.

#### Statistical analysis

Common contaminants were filtered, and proteins with at least 2 peptides were further considered. Fold change (FC) was calculated by median values for both conditions. If either of the conditions reported the absence of intensities for all replicates, a fold change of 100 or 0.1 was assigned. A two-sided *t*-test (assuming equal variances) was performed if more than 60% of the replicates had quantitative values. If a protein was exclusively present in only one condition, a *p*-value of 0.00001 was assigned. In case neither had more than 60% of values present across all replicates, a *p*-value of 1 was assigned. Benjamini-Hochberg (FDR) correction for multiple testing was applied to all *p*-values, including assigned values (1 and 0.00001). For the proteome dataset, proteins with FC ≥ 2 or ≤0.5 and adjusted *p*-value ≤ 0.05 were considered significant.

### Statistics and reproducibility

All experiments undergoing statistical analysis included at least three or more biological replicates with the number of repetitions as indicated in the figure legends. Statistical analyses were performed with Prism v9 software (GraphPad). Statistical tests were applied as indicated in the figure legends. All values are represented as mean ± SEM unless otherwise stated. *P* values were considered significant with *p* < 0.05. No blinding of mice or samples was conducted. No statistical methods were used to determine the sample size.

### Reporting summary

Further information on research design is available in the Nature Portfolio Reporting Summary linked to this article.

## Supplementary information

Supplementary Information

Reporting Summary

## Data Availability

All data generated and analyzed during this research are available as follows: The mass spectrometry proteomics data have been deposited to the ProteomeXchange Consortium (http://proteomecentral.proteomexchange.org) via the jPOST partner repository (https://repository.jpostdb.org/ PMID:27899654) with the dataset identifiers PXD055979 (ProteomeXchange) and JPST003367 (jPOST)^52^. Gating strategy and unedited Western blots are in the Supplementary Information PDF, Supplementary Figs. 5 and 6.
