# Supplementary material for: Prohibitin 2 is a key regulator of T cell proliferation, differentiation, and effector functions in vivo
Source: Commun Biol. 2026 Jul 20;9:989. doi: 10.1038/s42003-026-10522-3 (PMC13385934; doi:10.1038/s42003-026-10522-3)
Supplement: Supplementary file 1 — Supplementary Information [file 42003_2026_10522_MOESM1_ESM.pdf]

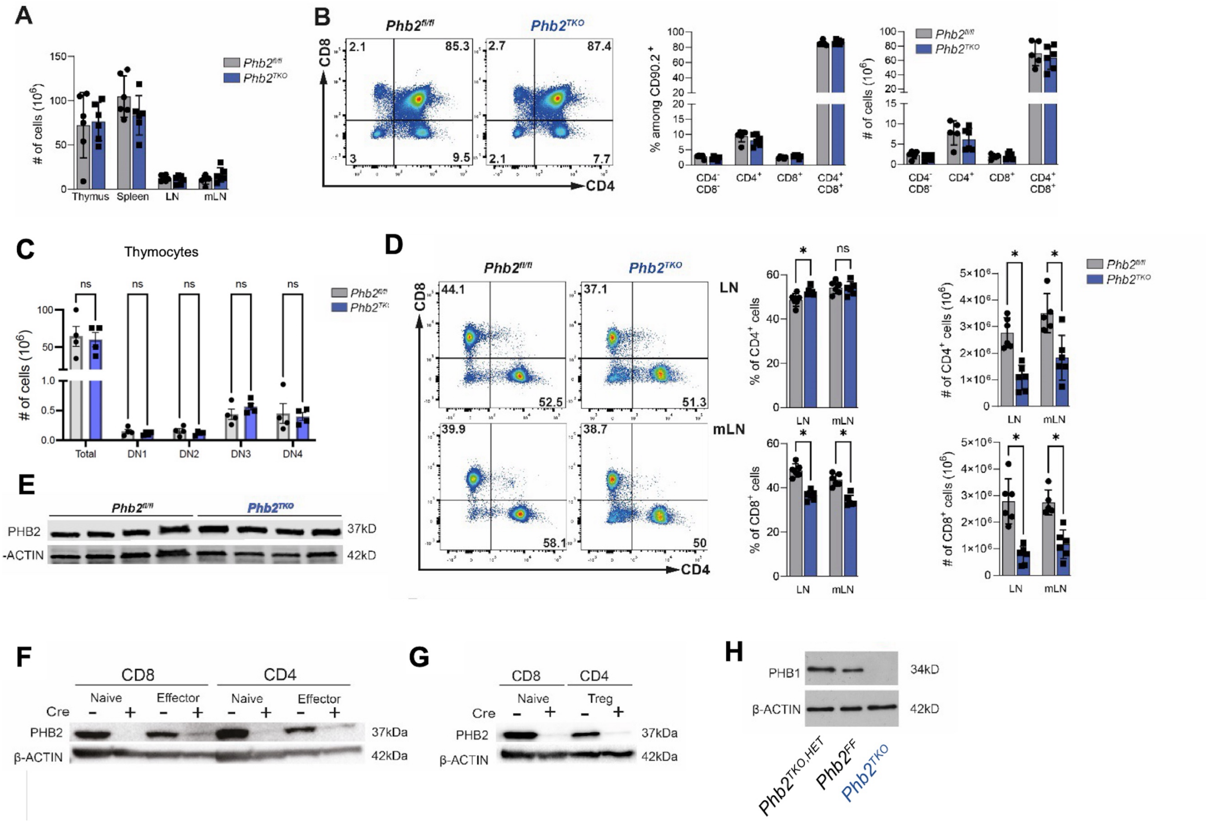

Figure S1: Prohibitin 2 is essential for T cell homeostasis *in vivo.*

(**A**) Bar graph depicting the total cell count of thymus, spleen, lymph nodes (LN), and mesenteric LN (mLN)

(**B**) Flow cytometric (left) and statistical (right) analysis of live CD90⁺ CD4⁺ and CD8⁺ thymocytes

(**C**) CD19⁻CD11b⁻, CD4⁻CD8⁻ double-negative (DN) T cell precursors in the thymus were analyzed by flow cytometry into DN1 (CD44⁺CD25⁻), DN2 (CD44⁺CD25⁺), DN3 (CD44⁻CD25⁺), and DN4 (CD44⁻CD25⁻) subpopulations based on CD25 and CD44 expression. 4/4 mice were used per experiment and repeated twice. Ns = non-significant

(**D**) Flow cytometric and statistical analysis of live, TCRβ⁺ CD4⁺ and CD8⁺ T cells in LN and mLN

(**E**) Representative Western blot analysis of PHB2protein expression in CD4⁺ thymic T cells, isolated by magnetic cell sorting (MACS)

(**F**) Western blot analysis of PHB2 in FACS sorted splenic naïve CD4⁺ (CD62L⁺ CD44^{low}), CD4⁺ effector (CD62L⁻ CD44^{high}), CD8⁺ naïve (CD62L⁺ CD44^{low}) and pooled CD8⁺ effector and central memory (CD62L⁻ CD44^{high} and CD62L⁺ CD44^{high}) T cells

(**G**) Western blot analysis of PHB2 of flow cytometry sorted splenic Treg cells (CD4⁺, CD25⁺), and naïve CD8⁺ (CD62L⁺ CD44^{low}) T cells

(**H**) Western Blot analysis of PHB1 in MACS sorted CD4⁺ splenic T cells

Data are representative of at least three independent experiments with n= 5-6 mice per genotype/experiment. Analyses were performed on 8-12-week-old mice. Bar graphs show means +/- SDs and single values (A, C, F). Statistical significance was calculated using an unpaired two-tailed t-test with Holm-Šidák correction for multiple comparisons. *p<0,05.

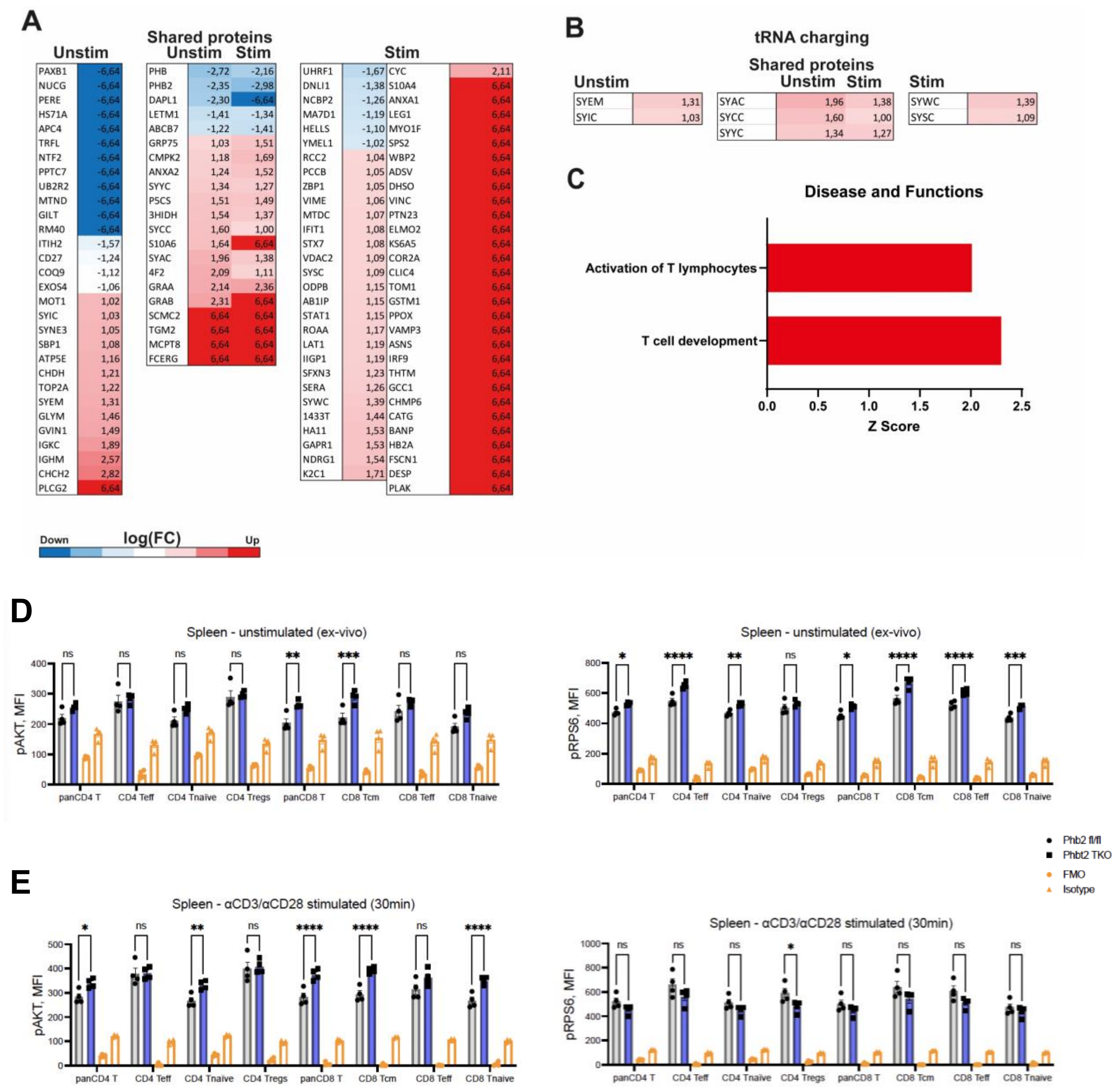

Figure S2: PHB2 regulates proteins that are essential for proliferation.

(**A**) Fold change list of significantly regulated peptides in unstimulated and α-CD3/α-CD28 stimulated control and PHB2-deficient naïve CD4^{+} T cells, with n=5 pooled samples per genotype

(**B**) Fold change of significant regulated tRNA transferase peptides

(**C**) Ingenuity Pathways Analysis of significantly regulated (Z Score ≥2) Diseases and Functions

(**D**) Bar graphs of MFI of p-AKT and p-RP6S levels of ex vivo (unstimulated) T cell subsets from $Phb2^{TKO}$ and control cells.

(**E**) Bar graphs of MFI of p-AKT and p-RP6S levels after 30min aCD3/aCD28 stimulated T cell subsets from $Phb2^{TKO}$ and control cells

(**D and E**) Data are representative of two independent experiments with n= 4-5 mice per genotype/experiment. Analyses were performed on 8-12-week-old mice. Bar graphs show means +/- SDs and single values. Statistical significance was calculated using an unpaired two-tailed t-test with Holm-Šidák correction for multiple comparisons. *p<0,05

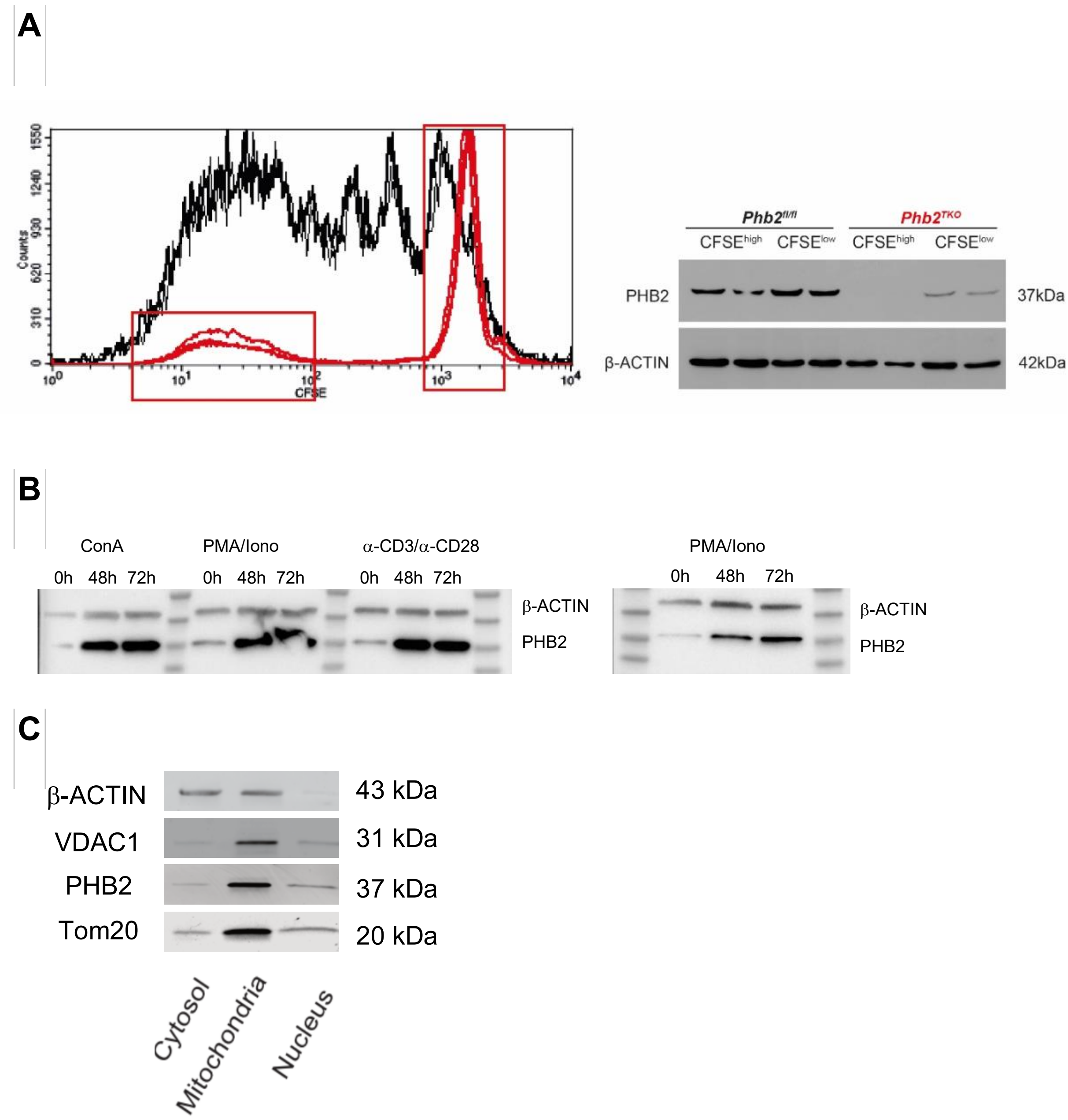

Figure S3: PHB2-expressing T cells outproliferate PHB2-deficient T cells.
(**A**)Western blot analysis (right) of PHB2 protein expression in CD4⁺ T cells, which were sorted by flow cytometry based on status: CFSE^{low}, proliferating cells, and CFSE^{high} for non-proliferating CD4⁺ T cells. Cells were stimulated *in vitro* with α-CD3/α-CD28 for 4 days
(**B**) MACS-purified wild-type T cells were left untreated or were activated with different stimuli and were harvested 48 and 72h later. PHB2 and Actin protein levels were determined by Western blot analysis. Representative data from 2 independent experiments. PHB2: 37kDa, Actin: 42kDa)
(**C**) Western blot analysis of subcellular localization (cytosolic, mitochondrial, and nuclear) of PHB2 in MACS-purified wild-type T cells. Fraction purity was validated using compartment-specific markers: β-actin (cytosol, 43 kDa), VDAC1 (mitochondria, 31 kDa), and Tom20 (mitochondria, 20 kDa).

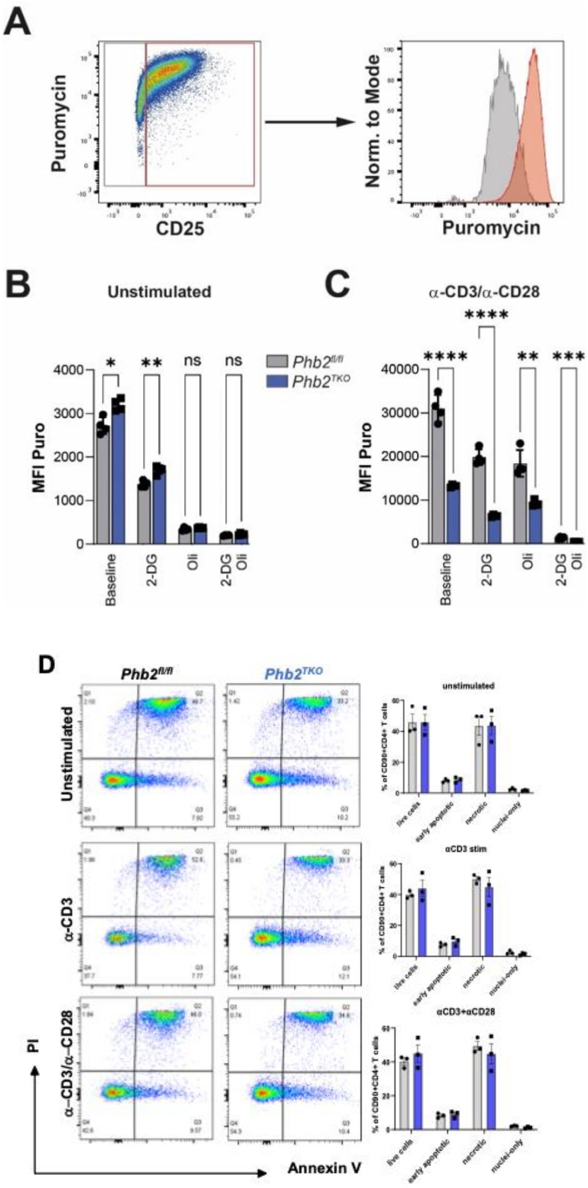

Figure S4: CD25⁺ T cells incorporate more puromycin.

(**A**) Flow cytometric analysis of Puromycin and CD25 co-expression (orange) on naive CD4⁺ T cells stimulated for 24h with α-CD3/α-CD28 (n=4)

(**B, C**) Mean fluorescence intensity (MFI) of puromycin in CD4⁺ naïve T cells unstimulated (**B**) and 24h α-CD3/α-CD28 stimulated (C). Cells were incubated with metabolic inhibitors Oligomycon (Oli) and 2DG, with at least n=4 mice/genotype

(**D**) Flow cytometry-based apoptotic analysis with propidium Iodide and annexin V staining of CD90+CD4+ T cells following overnight incubation in full RPMI medium, at steady state, with anti-CD3 stimulation and anti-CD3/anti-CD28 stimulation (n = 4)

The data are representative of three independent experiments. Analyses were performed on 8-12-week-old mice. Bar graphs show means +/- SDs and single values (C-I). Statistical significance was calculated using an unpaired two-tailed t-test with Holm-Šidák correction for multiple comparisons. *p<0.05, **p≤0.01, ***p≤0.001, ****p≤0.0001

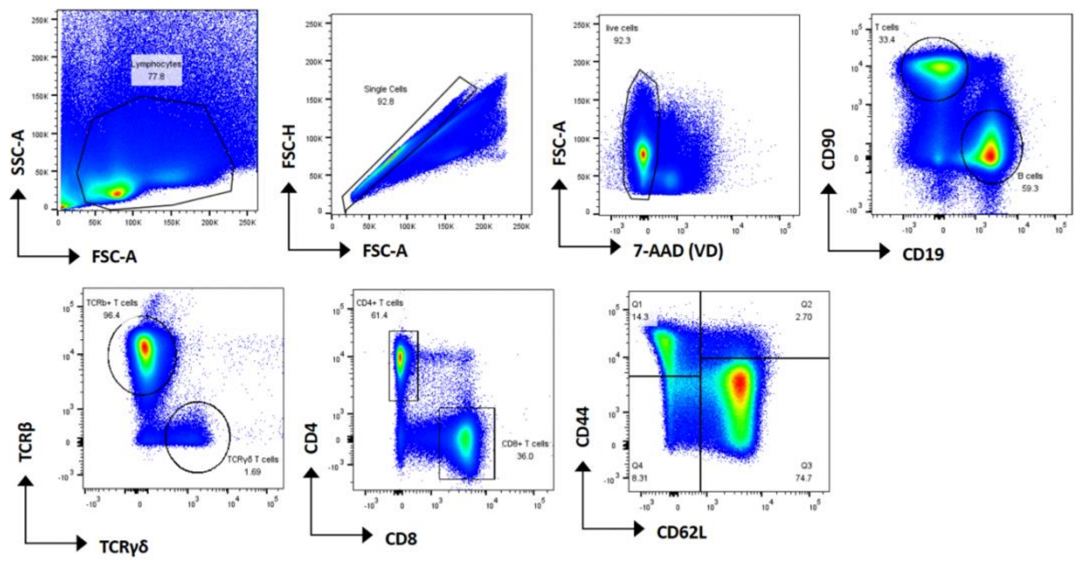

Figure S5. Gating strategy for splenic T cell subsets.

Splenocytes were first gated on lymphocytes based on forward scatter (FSC-A) and side scatter (SSC-A) parameters. Doublets were excluded using FSC-A versus FSC-H, followed by exclusion of dead cells using 7-AAD viability dye. B cells were excluded by gating out $CD19^+$ cells, and $CD90^+$ T cells were selected. T cells were further subdivided into $TCR\beta^+$ and $TCR\gamma\delta^+$ populations. Within the $TCR\beta^+$ compartment, $CD4^+$ and $CD8^+$ T cells were identified. Naive and antigen-experienced subsets were defined based on CD62L and CD44 expression. Representative gating plots are shown.

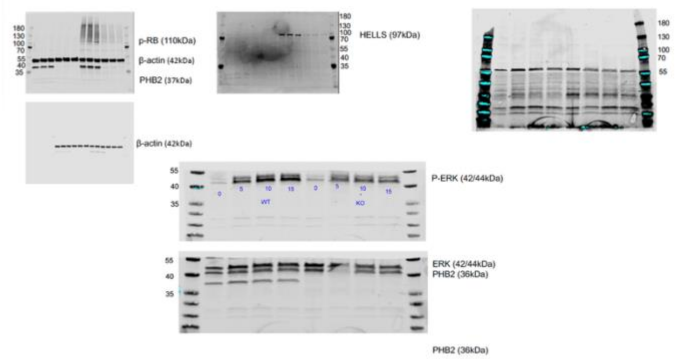
180
130
100
70
55
40
35
p-RB (110kDa)
β-actin (42kDa)
PHB2 (37kDa)
HELLS (97kDa)
β-actin (42kDa)
P-ERK (42/44kDa)
WT
KO
ERK (42/44kDa)
PHB2 (36kDa)
PHB2 (36kDa)

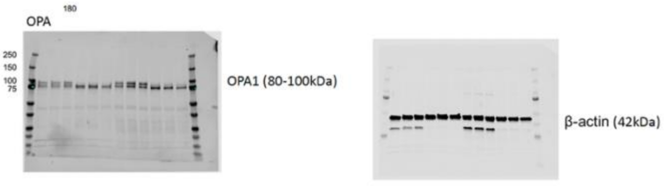
OPA
180
250
150
100
75
OPA1 (80-100kDa)
β-actin (42kDa)

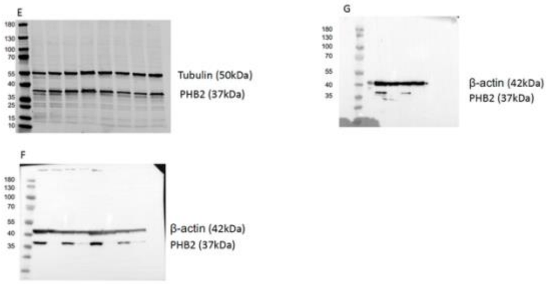
E
Tubulin (50kDa)
PHB2 (37kDa)
G
β-actin (42kDa)
PHB2 (37kDa)
F
β-actin (42kDa)
PHB2 (37kDa)

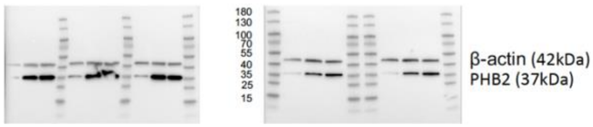
180
130
100
70
55
40
35
25
15
β-actin (42kDa)
PHB2 (37kDa)

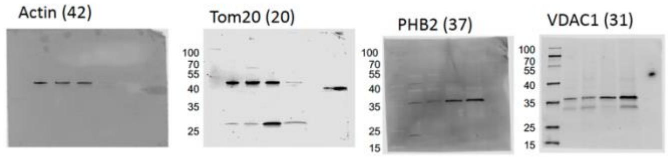
Actin (42)
Tom20 (20)
PHB2 (37)
VDAC1 (31)

Table S1: Proliferation associated proteins in PHB2 deficient T cells

Protein Name	Condition	Role described	Citations
HELLS	Stim	Essential for proliferation of peripheral T cells	T. M. Geiman and K. Muegge 2000
DNLI1	Stim	Immunodeficiency	P. Maffucci 2018, T. R. L. Howes and A.E. Tomkinson 2012
UHRF1	Stim	tumor progression, tumor severity, regulation of colonic T reg cell proliferation	S. C. Wu et al. 2022, Y. Obata et al. 2014
RCC2	Stim	progression in the metaphase	D. Papini et al. 2015
NDRG1	Stim	associated with T cell anergy induction	Y.M. Oh et al. 2015
LEG1	Stim	apoptosis and cell cycle bock at G1	T. Lin et al. 2014
BANP	Stim	activation of p53 and subsequent p21	R. Kaul 2003, P. Bao 2023
Paxbp1	Unstim	Survival of T cells, checkpoint for cell growth, induction of p53	W. Li 2023, S. Zhou 2021
NCBP2	Stim	expression positively correlates with tumor growth	H. Bu et al. 2022

Figure 1

Sample:	B cells | Freq	B cells | Freq	T cells/CD4 |	T cells/CD4 |
Spleen Naive EM,2f,CM_Tube_0	51,1	51,1	23,5	59,1
Spleen Naive EM,2f,CM_Tube_0	58,4	58,4	19,4	60,2
Spleen Naive EM,2f,CM_Tube_0	59,6	59,6	20	62,6
Spleen Naive EM,2f,CM_Tube_0	56,2	56,2	20,3	59
Spleen Naive EM,2f,CM_Tube_0	74,2	74,2	6,76	58,5
Spleen Naive EM,2f,CM_Tube_0	74,1	74,1	9,55	71
Spleen Naive EM,2f,CM_Tube_0	67,9	67,9	11,1	65,1
Spleen Naive EM,2f,CM_Tube_0	73,3	73,3	9,63	70,4
Mean	64,3	64,3	15	63,2
SD	9,14	9,14	6,4	5,1

	28,105		12,925	
	35,624		11,834	
	32,184		10,8	
	15,736		5,684	
	23,744		2,1632	
	22,971		2,9605	
	27,16		4,44	
	0		0	

	Phb2$^{fl/fl}$			
12,7	13,5	12,6	13,4	9,72

	Phb2$^{fl/fl}$			
1,4544	1,4795	1,6287	1,5174	0,1904

T cells/CD4/E	T cells/CD4/E	T cells/CD4/Naive T c	T cells/CD4/Naive T cell	T cells/CD8 |	T cells/CD8 |
3,88	16,5	17,4	74,2	15,2	38,2
3,06	15,8	14,5	75	11,9	37
3,67	18,4	14,6	73	11,2	35
3,37	16,6	15,3	75,1	13,2	38,3
0,68	10	4,64	68,6	4,48	38,8
1,09	11,5	6,78	71	3,54	26,3
1,18	10,7	8,11	73,4	5,41	31,8
1,23	12,7	7,16	74,3	3,52	25,7
2,27	14	11,1	73,1	8,56	33,9
1,34	3,17	4,87	2,24	4,79	5,37
2,134		9,57		8,36	
1,8666		8,845		7,259	
1,9818		7,884		6,048	
0,9436		4,284		3,696	
0,2176		1,4848		1,4336	
0,3379		2,1018		1,0974	
0,472		3,244		2,164	
0		0		0	

Phb2^{TKO}

6,97	8,91	8,19	% Tregs

Phb2^{TKO}

0,224	0,3131	0,32	# Tregs

T cells/CD8/C	T cells/CD8/C	T cells/CD8/E	T cells/CD8/E	T cells/CD8/N	T cells/CD8/N	Tcell | Freq. c
2,22	14,6	0,66	4,32	11,8	77,8	41,2
1,98	16,6	0,81	6,81	8,72	73,2	33,4
1,65	14,8	0,85	7,57	8,34	74,5	33
2,57	19,5	0,63	4,79	9,65	73,1	35,4
0,21	4,7	2,19	48,8	1,72	38,5	15
0,29	8,26	0,27	7,55	2,77	78,2	17,6
0,44	8,12	1,01	18,6	3,48	64,3	23,2
0,27	7,77	0,38	10,9	2,61	74,2	17,2
1,2	11,8	0,85	13,7	6,14	69,2	27
1	5,24	0,59	14,9	3,9	13,1	9,94
1,221		0,363		6,49		22,66
1,2078		0,4941		5,3192		20,374
0,891		0,459		4,5036		17,82
0,7196		0,1764		2,702		9,912
0,0672		0,7008		0,5504		4,8
0,0899		0,0837		0,8587		5,456
0,176		0,404		1,392		9,28
0		0		0		0

Tcell | Freq. c	Sample:	R1/R2/E3/live	R1/R2/E3/live	R1/R2/E3/live	R1/R2/E3/live cells/T cell TC
41,2	Spleen Naive	51	51	40,1	40,1
33,4	Spleen Naive	58,3	58,3	32,2	32,2
33	Spleen Naive	59,5	59,5	32,1	32,1
35,4	Spleen Naive	56,1	56,1	34,7	34,7
15	Spleen Naive	74	74	11,8	11,8
17,6	Spleen Naive	74	74	13,9	13,9
23,2	Spleen Naive	67,8	67,8	17,5	17,5
17,2	Spleen Naive	73,2	73,2	13,9	13,9
27	Mean	64,2	64,2	24,5	24,5
9,94	SD	9,12	9,12	11,3	11,3
		28,05		22,055	
		35,563		19,642	
		32,13		17,334	
		15,708		9,716	
		23,68		3,776	
		22,94		4,309	
		27,12		7	
		0		0	

EAE

	day				
		11	0	0	0
		12	0	0	0
		13	0	1,5	0
		14	0	1,5	0,5
		15	0	3	3
		17	0	3,5	3
		18	0	4	4
		19	0	4	4
		22	0	3	2,5
		24	0	2	2
		26	0	1,5	2

BM chimeras EAE

0	0	0	0
1	0	0	0
2	0	0	0
3	0	0	0
4	0	0	0
5	0	0	0
6	0	0	0
7	0	0	0
8	0	0	0
9	0	0	0
10	0	0	0
11	0	0	0
12	0,5	0	0
13	3	0,5	0
14	3,5	2	0
15	4	3	1,5

Foxp3Cre

G

Thymus	170000000	210000000	135000000
Spleen	54000000	31000000	40000000
LN	3400000	5000000	7200000

Foxp3Cre animals

Sample:		Tregs | Freq. of Liv	Tregs | Freq. c	Tregs | Geom
Spleen FOPX3 IC_Tube_001.fcs		3,03	12,7	1436
Spleen FOPX3 IC_Tube_002.fcs		2,69	13,5	1443
Spleen FOPX3 IC_Tube_003.fcs		2,67	12,6	1152
Spleen FOPX3 IC_Tube_004.fcs		2,81	13,4	1267
Spleen FOPX3 IC_Tube_005.fcs		0,68	9,72	1075
Spleen FOPX3 IC_Tube_006.fcs		0,7	6,97	1258
Spleen FOPX3 IC_Tube_007.fcs		1,01	8,91	1675
Spleen FOPX3 IC_Tube_008.fcs		0,8	8,19	1374
Mean		1,8	10,7	1335
SD		1,08	2,59	189
WT Spl		Phbt FoxP3 Cre	WT Thy	Phbt FoxP3 Cr
	54000000	27000000	170000000	24000000
	31000000	60000000	210000000	57000000
	40000000	25000000	135000000	5400000

H		ctr	
Spleen	**7,55**	**8,45**	**6,61**
LN	**7,52**	**11**	**6,89**
**I**			
Naive	74,7	73,4	73
Effector	13,9	15	15,6
Naive	59,4	65,2	60,6
Effector	8,17	7,05	12,3
CentralMemory	27,6	22,3	22,2
J			
Naive	86,6	82,9	84
Effector	5	7,19	6,59
I			
Naive	60,1	62,1	60,9
Effector	2,13	2,17	4,02
CentralMemory	33,4	30,5	30,3

	ctrl.				
0		0	0	0	0
0		0	2	0	0
0		0	3,5	2	1,5
0		0	4	3	3,5
0		1,5	4	4	4
0		2,5	4	4	3,5
0		3,5	4	4	3
0		3,5	4	4	3
0		2,5	3,5	3,5	2
0		2	3	3	2
0		2	3	2,5	1,5

CD90.1/CD90.2 %

Control			
36,7	22,7	33	4,12

CD40L CNS

Control			
34,4	36,7	36,7	6,46

24000000	57000000	5400000
27000000	60000000	25000000
51000000	63000000	32000000

etric Mean (Foxp3)

WT LN	Phbt FoxP3 Cre			
3400000	51000000			
5000000	63000000			
7200000	32000000			
			Phb2tregko	
**6,07**	**5,14**	**4,83**	0,44	0,77
**3,18**	**3,69**	**4,35**	10,3	5,98
73,5	67,3	10,7	7,54	13,8
11,9	18	79	82,9	70
47,4	45,3	8,19	9,98	9,37
22,7	20,1	79,1	70,9	72
24,2	31,1	3,13	8,89	16,8
86,3	84,4	30	25,6	47,6
3,79	4,79	52,3	59	33,4
72,4	70,6	20,6	20,5	29
1,86	2,19	32,1	30,1	32,4
20,6	23,5	41,7	43	34,3

phbt

0	0	0	0	0	0	0
0	0	0	0	0	0	0
0	0	0	0	0	0	0
0	0	0	0	0	0	0
0	0	0	0	0	0	0
0	0	0	0	0	0	0
0	0	0	0	0	0	0
0	0	0	0	0	0	0
0	0	0	0	0	0	0
0	0	0	0	0	0	0
0	0	0	0	0	0	0

$Phb2^{TKO}$

2,7	2,54

$Phb2^{TKO}$

7,6	9,5

0,55	0,79	1,21
1,78	1,62	1,79

15,6
69

6,49
80,2
10,9

43
38,3

25,8
39
31,1

Max score	Phbt2$^{fl/fl}$	Phb2^{TKO}	AUC	Phbt2$^{fl/fl}$
	4	0		0
	4	0		36
	3,5	0		31,75
	4	0		0
	4	0		26,75
	3	0		48,25
	0	0		43,5
	0			34

Phb2^{TKO}
0
0
0
0
0
0
0

IL-2 (pg/ml)

	Control		
Unstimulated	2,198	39,43	21,258
Stimulated	1243,771	1086,914	586,452

	$Phb2^{TKO}$	
25,164	0	46,384
2405,694	1825,916	1662,548

OPA processing

	Phb2$^{fl/fl}$			
Control	0,8849372	0,8439425	0,6273187	0,1222798
Stimulated	0,8143608	1,2532943	1,1089552	0,1368

TMRE/MTG

Sample:	R1/R2/Live ce	R1/R2/Live cells/T cells/CD4 T cells/CD25+ | Geometric
MTG Stim_Tube_002.fcs	29119	37100
MTG Stim_Tube_003.fcs	36064	41611
MTG Stim_Tube_004.fcs	32387	39710
MTG Stim_Tube_005.fcs	34477	37142
MTG Stim_Tube_006.fcs	44349	47416
MTG Stim_Tube_007.fcs	38028	47587
MTG Stim_Tube_008.fcs	37778	44789

ROS

Sample:	CD4 T cells	CD4 T cells/CD25+
ROS Menadione_Tube_(	332	412
ROS Menadione_Tube_(	494	562
ROS Menadione_Tube_(	645	695
ROS Menadione_Tube_(	394	454
ROS Menadione_Tube_(	694	680
ROS Menadione_Tube_(	994	1056
ROS Menadione_Tube_(	577	711
ROS Menadione_Tube_(	570	767
ROS Stim_Tube_001.fcs	1237	1312
ROS Stim_Tube_002.fcs	1240	1359
ROS Stim_Tube_003.fcs	1201	1315
ROS Stim_Tube_004.fcs	1209	1280
ROS Stim_Tube_005.fcs	1068	1089
ROS Stim_Tube_006.fcs	843	892
ROS Stim_Tube_007.fcs	1054	1024
ROS Stim_Tube_008.fcs	1134	1169
ROS Unsti_Tube_001.fcs	1165	321
ROS Unsti_Tube_002.fcs	1182	298
ROS Unsti_Tube_003.fcs	1232	610
ROS Unsti_Tube_004.fcs	1235	295
ROS Unsti_Tube_005.fcs	1306	476
ROS Unsti_Tube_006.fcs	1352	486
ROS Unsti_Tube_007.fcs	1503	385
ROS Unsti_Tube_008.fcs	867	243

Mean	980	745
SD	333	371

Flow seahorse

r	Lymphocytes/S	Lymphocytes/S	Lymphocytes/Single Cells/live /CD90+ T cells/
Phbt2 3rd_aKOS6.fcs	942	368	0,25
Phbt2 3rd_aKOS7.fcs	926	365	0,26
Phbt2 3rd_aKOS8.fcs	1120	445	0,27
Phbt2 3rd_awtS1.fcs	1978	963	0,31
Phbt2 3rd_awtS2.fcs	2523	1173	0,31
Phbt2 3rd_awtS3.fcs	2573	1225	0,32
Phbt2 3rd_awtS4.fcs	2650	1268	0,32
Phbt2 3rd_koS6.fcs	1664	741	0,3
Phbt2 3rd_koS7.fcs	1819	817	0,3
Phbt2 3rd_koS8.fcs	1833	820	0,3
Phbt2 3rd_wtS1.fcs	891	363	0,26
Phbt2 3rd_wtS2.fcs	1507	633	0,28
Phbt2 3rd_wtS3.fcs	1587	683	0,29
Phbt2 3rd_wtS4.fcs	1674	718	0,29
Mean	1734	756	0,29
SD	644	301	0,025

Phb2^{TKO}

0,1333333	0,1488801
0,1604938	0,1878453

: Mean (MTG)

MTG Unsti_T	21561	24354
MTG Unsti_T	20736	17205
MTG Unsti_T	24165	21494
MTG Unsti_T	24551	22712
MTG Unsti_T	24937	23074
MTG Unsti_T	23260	23417
Mean	30109	32893
SD	7560	11051

C

%Thymus

Sample:	Group	R1/R2/R3/VD-/CD1<br>**CD8- CD4+ of VD-**
Thymus_1.fcs	Phb2$^{fl/fl}$	5,87
Thymus_2.fcs	Phb2$^{fl/fl}$	8,11
Thymus_3.fcs	Phb2$^{fl/fl}$	8,41
Thymus_4.fcs	Phb2$^{fl/fl}$	8,3
Thymus_5.fcs	Phb2^{T-KO}	7,48
Thymus_6.fcs	Phb2^{T-KO}	8,12
Thymus_7.fcs	Phb2^{T-KO}	8,64
Thymus_8.fcs	Phb2^{T-KO}	8,99
Mean		7,99
SD		0,96
T test		0,085599737

#Thymus

Sample:	Group	DN1
Thymus_1.fcs	Phb2$^{fl/fl}$	0,08004
Thymus_2.fcs	Phb2$^{fl/fl}$	0,24
Thymus_3.fcs	Phb2$^{fl/fl}$	0,132
Thymus_4.fcs	Phb2$^{fl/fl}$	0,1425
Thymus_5.fcs	Phb2^{T-KO}	0,0836
Thymus_6.fcs	Phb2^{T-KO}	0,1368
Thymus_7.fcs	Phb2^{T-KO}	0,09
Thymus_8.fcs	Phb2^{T-KO}	0,1368
T test		0,260976105

B %

$CD4^- CD8^-$	3,04		3,12
$CD4^+$	9,54	10,6	9,86
$CD8^+$	2,12	2,6	2,79
$CD4^+ CD8^+$	85,3	82,7	84,2

A

Thymus	105	9	82,5
Spleen	135,2	89,6	104

LN	9,2	16	16,6
mLN	10,2	12	1,8

B count

	$CD4^{-}$ $CD8^{-}$	3,087
	$CD4^{+}$	9,6915
	$CD8^{+}$	2,1525
	$CD4^{+}$ $CD8^{+}$	86,73

D CD4 count			
	LN	2272400	3504000
	mLN	2876400	3024000
D CD8 count	LN	2346000	4192000
	mLN	2376600	2832000

R1/R2/R3/VD-/CD1 CD8+ CD4+ of VD-	R1/R2/R3/VD-/CD1 CD8+ CD4- of VD-	R1/R2/R3/VD-/CD: CD8- CD4- of VD-	R1/R2/R3/VD DN1 of DN	R1/R2/R3/VD DN1 of VD-	R1/R2/R3/VD DN2 of DN
76,7	2,14	1,73	13,2	0,23	10,3
85,1	2,9	2,11	11,5	0,24	11,9
86,5	2,81	1,64	12,4	0,2	13,8
84,4	2,94	1,73	14,7	0,25	16,7
87,1	2,2	2,58	8,48	0,22	15
87	2,67	1,78	10,1	0,18	9,2
86,3	2,82	1,92	9,49	0,18	8,38
85,7	2,73	1,99	9,18	0,18	8,66
84,9	2,65	1,93	11,1	0,21	11,7
3,42	0,31	0,3	2,18	0,029	3,14
0,127867725	0,151112534	0,175849515	0,01461125	0,03635236	0,18682419

DN2	DN3	DN4		Mio Thymus
0,06264	0,26448	0,19488		34,8
0,25	0,68	0,94		100
0,1518	0,4224	0,3762		66
0,1653	0,3762	0,2964		57
0,1482	0,4902	0,2584		38
0,1216	0,5928	0,494		76
0,08	0,465	0,325		50
0,1292	0,7144	0,5244		76
0,437098381	0,36734728	0,295031339		

3,07	1,51	2,85	2,59	2,87	2,47
10,4	5,77	10,8	8,57	10,1	9,96
2,54	1,95	2,52	2,7	3,2	3,25
84	90,8	83,9	86,1	83,8	84,3

108	54	75	96	84	102
78,4	130,8	90,8	87,6	75,6	50

13,4	8,9	11,5	4,8	12,8	5,1
12,1	12	16,6	30	12,8	14,6
2,48325	3,2076	0,7884	2,0325	2,3808	2,3352
7,84575	10,8	3,0078	7,65	7,8624	8,2404
2,21925	2,646	1,0206	1,8	2,4768	2,604
66,99	87,588	47,412	59,925	79,008	68,208
3037800	3269600	2189400	2380500	571200	1093120
	3351700	3552000	4731000	3270000	1497600
3054400	3068600	1833400	2208000	344160	847360
	2541000	2436000	3535800	1992000	820480

R1/R2/R3/VD	R1/R2/R3/VD	R1/R2/R3/VD	R1/R2/R3/VD-/CD11b- CD19-/Q4: CD8- , CD4-/Q4: CD25- ,
**DN2 of VD-**	**DN3 of DN**	**DN3 of VD-**	**DN4 of DN**
0,18	44	0,76	33
0,25	32	0,68	45
0,23	39	0,64	35
0,29	38,3	0,66	30
0,39	50,2	1,29	26
0,16	43,9	0,78	37
0,16	48,2	0,93	34
0,17	47,4	0,94	35
0,23	42,9	0,83	34
0,081	6,08	0,22	5
0,41690976	0,0021788	0,02122339	0

1,81	1,39	2,17
5,65	6,62	7,71
1,88	2,83	2,72
90,7	89,2	87,4
72	57	48
85,6	118,8	82,8

15	10,5	15,6
12,8	19,8	7

2,4072	1,26
9,6798	3,9312
3,162	1,3104
81,906	63,072

530400	1725000	1113000	#	49,9	52,5	49,7	55,9
1839600	1510400	2118600	#	55,6	58,1	56,1	53,4
385050	1231500	800100	#	46,8	44,1	46	33,7
1423500	1040640	1298880	#	42,1	39,9	41,8	32,7

51,1	51,3	51,2	52,3	52,9
58,9	50	51,6	54,1	55,1
39,6	37,1	36,7	37,5	34,7
32,2	38,7	35,5	33,2	32,8

ap	Lymphocytes	Lymphocytes	Lymphocytes	Lymphocytes/Single Cells/CD4+ T cells/Q
Phbt2 3wt vs	2.36 %	54.4 %	6.89 %	36.3 %
Phbt2 3wt vs	3.87 %	50.6 %	6.18 %	39.4 %
Phbt2 3wt vs	3.40 %	31.4 %	9.16 %	56.0 %
Phbt2 3wt vs	2.11 %	47.6 %	8.36 %	41.9 %
Phbt2 3wt vs	1.66 %	47.0 %	8.85 %	42.5 %
Phbt2 3wt vs	2.18 %	49.6 %	6.97 %	41.2 %
Phbt2 3wt vs	1.85 %	45.9 %	9.59 %	42.7 %
Phbt2 3wt vs	1.97 %	52.5 %	7.77 %	37.7 %
Phbt2 3wt vs	2.10 %	49.7 %	7.93 %	40.3 %
Phbt2 3wt vs	1.66 %	55.0 %	6.71 %	36.6 %
Phbt2 3wt vs	2.08 %	54.9 %	6.66 %	36.4 %
Phbt2 3wt vs	2.03 %	53.4 %	6.66 %	37.9 %
Phbt2 3wt vs	1.13 %	44.7 %	9.82 %	44.4 %
Phbt2 3wt vs	1.70 %	46.2 %	10.5 %	41.6 %
Phbt2 3wt vs	1.64 %	44.6 %	9.03 %	44.7 %
Phbt2 3wt vs	0.75 %	34.4 %	10.4 %	54.4 %
Phbt2 3wt vs	0.45 %	33.3 %	12.1 %	54.2 %
Phbt2 3wt vs	1.41 %	33.2 %	10.2 %	55.3 %
Phbt2 3wt vs	3.54 %	46.7 %	6.48 %	43.2 %
Phbt2 3wt vs	2.63 %	45.1 %	7.22 %	45.1 %
Phbt2 3wt vs	17.9 %	29.9 %	6.09 %	46.1 %
Mean	2.78 %	45.2 %	8.27 %	43.7 %
SD	3.56 %	8.08 %	1.71 %	6.30 %

C

Baseline	34944	30126	31577	27489
2-DG	19527	18984	22496	18405
Oli	17261	16263	22976	17130
2-DGOli	1057	1440	1557	1110

B

Baseline	2974	2645	2734	2522
2-DG	1475	1314	1403	1314
Oli	391	342	307	319
2-DGOli	207	200	206	186

4: Annexin V- , PI- | Freq. of Parent

13284	13265	12844	13535
6332	6741	6318	6074
8986	10280	8887	8705
397	458	366	391

3059	3099	3273	3351
1743	1567	1782	1723
371	363	387	365
218	259	231	201

%Spleen		R1/R2/R3/VD pAKT	R1/R2/R3/VD pAKT
Sample:	Group	pan CD4 T	CD4 eff
Spleen-FMO_ISO_1FMO.fcs	$Phb2^{fl/fl}$	34,6	-3,65
Spleen-FMO_ISO_2FMO.fcs	$Phb2^{fl/fl}$	46,8	12,2
Spleen-FMO_ISO_3ISO.fcs	$Phb2^{fl/fl}$	125	103
Spleen-FMO_ISO_4ISO.fcs	$Phb2^{fl/fl}$	128	108
Spleen-FMO_ISO_5FMO.fcs	$Phb2^{T-KO}$	36	-10,8
Spleen-FMO_ISO_6FMO.fcs	$Phb2^{T-KO}$	42,1	0,28
Spleen-FMO_ISO_7ISO.fcs	$Phb2^{T-KO}$	119	88,9
Spleen-FMO_ISO_8ISO.fcs	$Phb2^{T-KO}$	116	85,7
Spleen-pAKT_1.fcs	$Phb2^{fl/fl}$	284	370
Spleen-pAKT_2.fcs	$Phb2^{fl/fl}$	264	358
Spleen-pAKT_3.fcs	$Phb2^{fl/fl}$	322	446
Spleen-pAKT_4.fcs	$Phb2^{fl/fl}$	263	338
Spleen-pAKT_5.fcs	$Phb2^{T-KO}$	361	407
Spleen-pAKT_6.fcs	$Phb2^{T-KO}$	319	364
Spleen-pAKT_7.fcs	$Phb2^{T-KO}$	350	397
Spleen-pAKT_8.fcs	$Phb2^{T-KO}$	327	357
Spleen-stim-FMO_ISO_1FMO.fcs	$Phb2^{fl/fl}$	93,7	47,5
Spleen-stim-FMO_ISO_2FMO.fcs	$Phb2^{fl/fl}$	83,6	41
Spleen-stim-FMO_ISO_3ISO.fcs	$Phb2^{fl/fl}$	174	143
Spleen-stim-FMO_ISO_4ISO.fcs	$Phb2^{fl/fl}$	139	101
Spleen-stim-FMO_ISO_5FMO.fcs	$Phb2^{T-KO}$	86,2	25,5
Spleen-stim-FMO_ISO_6FMO.fcs	$Phb2^{T-KO}$	90,7	21,8
Spleen-stim-FMO_ISO_7ISO.fcs	$Phb2^{T-KO}$	184	138
Spleen-stim-FMO_ISO_8ISO.fcs	$Phb2^{T-KO}$	173	144
Spleen-stim-pAKT_1.fcs	$Phb2^{fl/fl}$	202	243
Spleen-stim-pAKT_2.fcs	$Phb2^{fl/fl}$	205	260
Spleen-stim-pAKT_3.fcs	$Phb2^{fl/fl}$	257	333
Spleen-stim-pAKT_4.fcs	$Phb2^{fl/fl}$	216	264
Spleen-stim-pAKT_5.fcs	$Phb2^{T-KO}$	243	261
Spleen-stim-pAKT_6.fcs	$Phb2^{T-KO}$	254	293
Spleen-stim-pAKT_7.fcs	$Phb2^{T-KO}$	271	295
Spleen-stim-pAKT_8.fcs	$Phb2^{T-KO}$	256	284

R1/R2/R3/VD	R1/R2/R3/VD	R1/R2/R3/VD	R1/R2/R3/VD	R1/R2/R3/VD	R1/R2/R3/VD	XXX
**pAKT**	**pAKT**	**pAKT**	**pAKT**	**pAKT**	**pAKT**	
**CD4 naive**	**CD4 Tregs**	**pan CD8 T**	**CD8 CM**	**CD8 eff**	**CD8 naive**	
37,7	18,5	4,59	-4,18	-5,77	6,12	
50,2	33,8	17,6	9,29	2,94	18,8	
127	101	108	119	111	106	
130	105	110	119	112	108	
42,2	15,6	3,93	-2,04	-0,44	1,4	
43,7	25,1	2,61	-3,16	-3,77	0,81	
122	91,2	98,3	116	102	98,8	
118	90,3	94,4	111	102	93,5	
271	398	288	296	323	273	
248	370	255	270	283	242	
303	476	327	335	367	306	
249	350	261	274	283	249	
350	444	399	401	408	362	
312	387	349	378	326	330	
343	418	389	409	366	364	
321	386	363	389	345	345	
98,5	66,3	60,8	49,2	43,7	63,5	
89,7	58,8	52,1	40,6	30,8	54,5	
178	137	150	154	144	151	
144	107	113	111	106	114	
101	61,7	55,1	40,9	38,5	56,6	
95,7	66,4	49,3	38,6	26,3	52,3	
188	149	161	176	151	163	
176	144	163	177	166	164	
195	264	186	204	219	175	
196	275	187	204	212	177	
246	352	245	263	297	226	
208	269	197	214	243	187	
233	288	256	261	280	214	
249	300	259	295	256	236	
266	311	283	311	278	255	
252	291	260	292	276	240	

			R1/R2/R3/VD
**%Spleen**			**pRPS6**
**Sample:**		**Group**	**pan CD4 T**
Spleen-FMO_ISO_1FMO.fcs	Stimulated	$Phb2^{fl/fl}$	34,6
Spleen-FMO_ISO_2FMO.fcs	Stimulated	$Phb2^{fl/fl}$	46,8
Spleen-FMO_ISO_3ISO.fcs	Stimulated	$Phb2^{fl/fl}$	125
Spleen-FMO_ISO_4ISO.fcs	Stimulated	$Phb2^{fl/fl}$	128
Spleen-FMO_ISO_5FMO.fcs	Stimulated	$Phb2^{T-KO}$	36
Spleen-FMO_ISO_6FMO.fcs	Stimulated	$Phb2^{T-KO}$	42,1
Spleen-FMO_ISO_7ISO.fcs	Stimulated	$Phb2^{T-KO}$	119
Spleen-FMO_ISO_8ISO.fcs	Stimulated	$Phb2^{T-KO}$	116
Spleen-pRPS6_1.fcs	Stimulated	$Phb2^{fl/fl}$	487
Spleen-pRPS6_2.fcs	Stimulated	$Phb2^{fl/fl}$	528
Spleen-pRPS6_3.fcs	Stimulated	$Phb2^{fl/fl}$	602
Spleen-pRPS6_4.fcs	Stimulated	$Phb2^{fl/fl}$	473
Spleen-pRPS6_5.fcs	Stimulated	$Phb2^{T-KO}$	477
Spleen-pRPS6_6.fcs	Stimulated	$Phb2^{T-KO}$	466
Spleen-pRPS6_7.fcs	Stimulated	$Phb2^{T-KO}$	475
Spleen-pRPS6_8.fcs	Stimulated	$Phb2^{T-KO}$	400
Spleen-stim-FMO_ISO_1FMO.fcs	Unstimulated	$Phb2^{fl/fl}$	93,7
Spleen-stim-FMO_ISO_2FMO.fcs	Unstimulated	$Phb2^{fl/fl}$	83,6
Spleen-stim-FMO_ISO_3ISO.fcs	Unstimulated	$Phb2^{fl/fl}$	174
Spleen-stim-FMO_ISO_4ISO.fcs	Unstimulated	$Phb2^{fl/fl}$	139
Spleen-stim-FMO_ISO_5FMO.fcs	Unstimulated	$Phb2^{T-KO}$	86,2
Spleen-stim-FMO_ISO_6FMO.fcs	Unstimulated	$Phb2^{T-KO}$	90,7
Spleen-stim-FMO_ISO_7ISO.fcs	Unstimulated	$Phb2^{T-KO}$	184
Spleen-stim-FMO_ISO_8ISO.fcs	Unstimulated	$Phb2^{T-KO}$	173
Spleen-stim-pRPS6_1.fcs	Unstimulated	$Phb2^{fl/fl}$	468
Spleen-stim-pRPS6_2.fcs	Unstimulated	$Phb2^{fl/fl}$	454
Spleen-stim-pRPS6_3.fcs	Unstimulated	$Phb2^{fl/fl}$	501
Spleen-stim-pRPS6_4.fcs	Unstimulated	$Phb2^{fl/fl}$	466
Spleen-stim-pRPS6_5.fcs	Unstimulated	$Phb2^{T-KO}$	537
Spleen-stim-pRPS6_6.fcs	Unstimulated	$Phb2^{T-KO}$	539
Spleen-stim-pRPS6_7.fcs	Unstimulated	$Phb2^{T-KO}$	520
Spleen-stim-pRPS6_8.fcs	Unstimulated	$Phb2^{T-KO}$	518

R1/R2/R3/VD<br>**pRPS6**<br>**CD4 eff**	R1/R2/R3/VD<br>**pRPS6**<br>**CD4 naive**	R1/R2/R3/VD<br>**pRPS6**<br>**CD4 Tregs**	R1/R2/R3/VD<br>**pRPS6**<br>**pan CD8 T**	R1/R2/R3/VD<br>**pRPS6**<br>**CD8 CM**	R1/R2/R3/VD<br>**pRPS6**<br>**CD8 eff**	R1/R2/R3/VD<br>**pRPS6**<br>**CD8 naive**
-3,65	37,7	18,5	4,59	-4,18	-5,77	6,12
12,2	50,2	33,8	17,6	9,29	2,94	18,8
103	127	101	108	119	111	106
108	130	105	110	119	112	108
-10,8	42,2	15,6	3,93	-2,04	-0,44	1,4
0,28	43,7	25,1	2,61	-3,16	-3,77	0,81
88,9	122	91,2	98,3	116	102	98,8
85,7	118	90,3	94,4	111	102	93,5
613	478	545	468	590	580	438
685	516	605	504	635	611	474
787	589	695	593	778	717	556
556	465	521	450	563	527	424
597	474	512	465	575	547	462
574	465	485	457	577	525	443
610	474	510	475	582	523	470
462	396	411	384	437	446	374
47,5	98,5	66,3	60,8	49,2	43,7	63,5
41	89,7	58,8	52,1	40,6	30,8	54,5
143	178	137	150	154	144	151
101	144	107	113	111	106	114
25,5	101	61,7	55,1	40,9	38,5	56,6
21,8	95,7	66,4	49,3	38,6	26,3	52,3
138	188	149	161	176	151	163
144	176	144	163	177	166	164
522	466	469	450	538	497	439
544	447	473	433	531	539	420
599	491	566	488	628	548	469
527	459	505	439	554	509	418
654	536	558	510	694	594	521
679	538	529	529	691	628	521
646	518	531	516	678	587	521
628	513	508	493	618	620	492

XXX

**in vitro B**		Phbt2fl/fl				Phbt
IL17	15,4	11,7	7,43	6,62	0,2	0,21
Foxp3		Phbt2fl/fl				Phbt
	54,4	55,4	54	50,3	20,3	17,6

**C**	CD45.1	CD45.2
	77,6	21,4
	70,2	28,3
	73,4	25,9
	68,7	30,8
	72,3	25,6
	60,8	38

			PHB2fl/fl			
**G**	ADeadcells	44,6	40,5	46,7	42,3	38,5
	BApoptoticc	8,33	7,44	8,75	9,26	12,5
	CPre-Apopt	0,91	0,78	0,85	0,97	0,88
	DLiveCells	46,2	51,3	43,7	47,5	48,1

2^{TKO}		
	0,27	0,16

2^{TKO}		
	20,9	23,3

PHB2^{TKO}		
29,9	28,9	30,2
9,48	7,56	7,92
0,9	0,61	0,93
59,7	62,9	60,9
