# Supplementary material for: Prohibitin 2 is a key regulator of T cell proliferation, differentiation, and effector functions in vivo
Source: Commun Biol. 2026 Jul 20;9:989. doi: 10.1038/s42003-026-10522-3 (PMC13385934; doi:10.1038/s42003-026-10522-3)
Supplement: Supplementary file 2 — Reporting Summary [file 42003_2026_10522_MOESM2_ESM.pdf]

nature portfolio

Corresponding author(s): COMMSBIO-24-7934A

Last updated by author(s): 01.06.2026

Reporting Summary

Nature Portfolio wishes to improve the reproducibility of the work that we publish. This form provides structure for consistency and transparency in reporting. For further information on Nature Portfolio policies, see our Editorial Policies and the Editorial Policy Checklist.

Statistics

For all statistical analyses, confirm that the following items are present in the figure legend, table legend, main text, or Methods section.

n/a	Confirmed	
☐	☒	The exact sample size (*n*) for each experimental group/condition, given as a discrete number and unit of measurement
☐	☒	A statement on whether measurements were taken from distinct samples or whether the same sample was measured repeatedly
☐	☒	The statistical test(s) used AND whether they are one- or two-sided<br>*Only common tests should be described solely by name; describe more complex techniques in the Methods section.*
☐	☒	A description of all covariates tested
☐	☒	A description of any assumptions or corrections, such as tests of normality and adjustment for multiple comparisons
☐	☒	A full description of the statistical parameters including central tendency (e.g. means) or other basic estimates (e.g. regression coefficient) AND variation (e.g. standard deviation) or associated estimates of uncertainty (e.g. confidence intervals)
☒	☐	For null hypothesis testing, the test statistic (e.g. *F*, *t*, *r*) with confidence intervals, effect sizes, degrees of freedom and *P* value noted<br>*Give P values as exact values whenever suitable.*
☒	☐	For Bayesian analysis, information on the choice of priors and Markov chain Monte Carlo settings
☒	☐	For hierarchical and complex designs, identification of the appropriate level for tests and full reporting of outcomes
☒	☐	Estimates of effect sizes (e.g. Cohen's *d*, Pearson's *r*), indicating how they were calculated

Our web collection on statistics for biologists contains articles on many of the points above.

Software and code

Policy information about availability of computer code

Data collection	Data collection Western Blot: Image Lab, Flow cyotmetry: Facs Diva Software (FACSCANTO II). Massspectrometry: Hystar. Plate Reader: i-control (Tecan)
Data analysis	Prism 8 software (GraphPad) was used for data analysis. We observed normal distribution. Comparison of two groups were calculated using unpaired two-tailed Student's t test for parametric and Mann-Whitney test or Wilcoxon test for nonparametric distributions. Multiple comparison was corrected for using the Holm- Si´da´ k method. For comparison of more than two groups, one-way ANOVA with Bonferroni's posthoc test or Kruskal-Wallis test was used. Data are represented as means +/ SEM or mean ± SD, as indicated. Statistical significance: *p value <0.05; **p value <0.01; ***p value <0.001; ****p < 0.0001). ELISA was analyzed with SoftMax Pro 5.4.1 (Molecular Devices Corporation) and flow cytometry data were analyzed using the FlowJo software 8.87 (FLOWJO, LLC data analysis software). No sample was excluded from analysis.<br>The description of code used for the analysis of the mass spectrometry data can be found in the MM section.

For manuscripts utilizing custom algorithms or software that are central to the research but not yet described in published literature, software must be made available to editors and reviewers. We strongly encourage code deposition in a community repository (e.g. GitHub). See the Nature Portfolio guidelines for submitting code & software for further information.

Data

Policy information about availability of data

All manuscripts must include a data availability statement. This statement should provide the following information, where applicable:

- Accession codes, unique identifiers, or web links for publicly available datasets
- A description of any restrictions on data availability
- For clinical datasets or third party data, please ensure that the statement adheres to our policy

This paper does not report original code.
Any additional information required to reanalyze the data reported in this paper is available from the lead contact upon request.

Research involving human participants, their data, or biological material

Policy information about studies with human participants or human data. See also policy information about sex, gender (identity/presentation), and sexual orientation and race, ethnicity and racism.

Reporting on sex and gender	*Use the terms sex (biological attribute) and gender (shaped by social and cultural circumstances) carefully in order to avoid confusing both terms. Indicate if findings apply to only one sex or gender; describe whether sex and gender were considered in study design; whether sex and/or gender was determined based on self-reporting or assigned and methods used. Provide in the source data disaggregated sex and gender data, where this information has been collected, and if consent has been obtained for sharing of individual-level data; provide overall numbers in this Reporting Summary. Please state if this information has not been collected. Report sex- and gender-based analyses where performed, justify reasons for lack of sex- and gender-based analysis.*
Reporting on race, ethnicity, or other socially relevant groupings	*Please specify the socially constructed or socially relevant categorization variable(s) used in your manuscript and explain why they were used. Please note that such variables should not be used as proxies for other socially constructed/relevant variables (for example, race or ethnicity should not be used as a proxy for socioeconomic status). Provide clear definitions of the relevant terms used, how they were provided (by the participants/respondents, the researchers, or third parties), and the method(s) used to classify people into the different categories (e.g. self-report, census or administrative data, social media data, etc.) Please provide details about how you controlled for confounding variables in your analyses.*
Population characteristics	*Describe the covariate-relevant population characteristics of the human research participants (e.g. age, genotypic information, past and current diagnosis and treatment categories). If you filled out the behavioural & social sciences study design questions and have nothing to add here, write "See above."*
Recruitment	*Describe how participants were recruited. Outline any potential self-selection bias or other biases that may be present and how these are likely to impact results.*
Ethics oversight	*Identify the organization(s) that approved the study protocol.*

Note that full information on the approval of the study protocol must also be provided in the manuscript.

Field-specific reporting

Please select the one below that is the best fit for your research. If you are not sure, read the appropriate sections before making your selection.

☒ Life sciences ☐ Behavioural & social sciences ☐ Ecological, evolutionary & environmental sciences

For a reference copy of the document with all sections, see nature.com/documents/nr-reporting-summary-flat.pdf

Life sciences study design

All studies must disclose on these points even when the disclosure is negative.

Sample size	no sample size was calculated was done
Data exclusions	No data were excluded
Replication	All attempts at replications were successful
Randomization	Mice were allocated according to genotypes to groups.
Blinding	Experimenters were not blinded due to planing the group allocation aswell as conduiting data collection happened by the same person.

Reporting for specific materials, systems and methods

We require information from authors about some types of materials, experimental systems and methods used in many studies. Here, indicate whether each material, system or method listed is relevant to your study. If you are not sure if a list item applies to your research, read the appropriate section before selecting a response.

Materials & experimental systems

n/a	Involved in the study
☐	☒ Antibodies
☒	☐ Eukaryotic cell lines
☒	☐ Palaeontology and archaeology
☐	☒ Animals and other organisms
☒	☐ Clinical data
☒	☐ Dual use research of concern
☒	☐ Plants

Methods

n/a	Involved in the study
☒	☐ ChIP-seq
☐	☒ Flow cytometry
☒	☐ MRI-based neuroimaging

Antibodies

Antibodies used	TCRb-Fitc (Biolegend #109205), TCRb-PE-Cy7 (Biolegend #109221), CD19-PE-Cy7 (Biolegend #115520), CD4-PerCP (Biolegend #100432), CD4-BV510 (Biolegend #100559), CD4-PE (Biolegend #100408), CD8-Pacifique Blue (Biolegend #100725), CD8-BV510 (Biolegend #100752), CD62L-APC (Biolegend #104412), CD44-PE (eBioscience #12-0441), CD44-Fitc (eBioscience #11-0441), CD25-Fitc (BD Biosciences #553072), , CD45.1-PE-Cy7 (Biolegend #110730), CD45.2-Fitc (eBioscience #11-0454), FVD-APC-eFl780 (eBioscience #65-0865), FVD-eFl506 (eBioscience #65-0866) and CD69-Fitc (eBioscience #11-0691). Ki67-APC (Biolegend #652405) and Foxp3-APC (eBioscience 17-5773) with the Bioscience™ Foxp3/Transcription Factor Staining Buffer Set (00-5523-00).
Validation	All antibodies were bought and used as per manufacturers instructions and parameters.

Animals and other research organisms

Policy information about studies involving animals; ARRIVE guidelines recommended for reporting animal research, and Sex and Gender in Research

Laboratory animals	The following Mus Musculs lines were used: B6-Phb2tm1Tlan Tg(Cd4-cre)1Cwi/Tarc , B6-Phb2tm1Tlan Foxp3tm1(cre)Saka/Tarc. Age ranged from 29d to 16 week old.
Wild animals	This study did not involve wild animals
Reporting on sex	Results apply to both sex as sex of animals was not considered in the study design, thus both genders were used indiscrimnately in experiments.
Field-collected samples	The study did not involve samples collected from the field.
Ethics oversight	All experiments were in accordance with the guidelines of the Translational Animal Research Center (TARC) of the University of Mainz and approved by the institutional committee on animal experimentation and the government of Rheinland-Pfalz (RLP).

Note that full information on the approval of the study protocol must also be provided in the manuscript.

Plants

Seed stocks	*Report on the source of all seed stocks or other plant material used. If applicable, state the seed stock centre and catalogue number. If plant specimens were collected from the field, describe the collection location, date and sampling procedures.*
Novel plant genotypes	*Describe the methods by which all novel plant genotypes were produced. This includes those generated by transgenic approaches, gene editing, chemical/radiation-based mutagenesis and hybridization. For transgenic lines, describe the transformation method, the number of independent lines analyzed and the generation upon which experiments were performed. For gene-edited lines, describe the editor used, the endogenous sequence targeted for editing, the targeting guide RNA sequence (if applicable) and how the editor was applied.*
Authentication	*Describe any authentication procedures for each seed stock used or novel genotype generated. Describe any experiments used to assess the effect of a mutation and, where applicable, how potential secondary effects (e.g. second site T-DNA insertions, mosiacism, off-target gene editing) were examined.*

Flow Cytometry

Plots

Confirm that:

☒ The axis labels state the marker and fluorochrome used (e.g. CD4-FITC).

☒ The axis scales are clearly visible. Include numbers along axes only for bottom left plot of group (a 'group' is an analysis of identical markers).

☒ All plots are contour plots with outliers or pseudocolor plots.

☒ A numerical value for number of cells or percentage (with statistics) is provided.

Methodology

Sample preparation	Single cell suspensions of thymus, spleen, lymph nodes and mesenteric lymph nodes were prepared by smashing organs through a 40µm filter in PBS supplemented with 2% fetal calf serum (FCS). Following, red blood cell lysis was conducted on spleenocytes.
Instrument	All cells were acquired on BD FACS Canto II
Software	Flow cytometric data was collected with the FACS Diva software. The data was analyzed using FlowJo v.10.
Cell population abundance	The purity of facs sorted cells was above 95% and was confirmed by sorting data and a re-analysis of the sorted cells after sorting
Gating strategy	Cells were pregated in an SSC/FSC gate. Then doublets were excluded.

☒ Tick this box to confirm that a figure exemplifying the gating strategy is provided in the Supplementary Information.
